# An enteroendocrine-microbial axis in the large intestine controls host metabolism

**DOI:** 10.21203/rs.3.rs-3112286/v1

**Published:** 2023-07-06

**Authors:** Shuai Tan, Jacobo Santolaya, Tiffany Freeney Wright, Qi Liu, Teppei Fujikawa, Sensen Chi, Colin P. Bergstrom, Adam Lopez, Qing Chen, Goncalo Dias do Vale, Jeffrey G. McDonald, Da Jia, Joel K. Elmquist, Luis Sifuentes-Dominguez, Ezra Burstein

**Affiliations:** 1 Department of Immunology, School of Basic Medical Sciences, Chongqing Medical University, Chongqing 400010, China; 2 Department of Internal Medicine, The University of Texas Southwestern Medical Center, Dallas, TX 75390, USA.; 3 Department of Pediatrics, The University of Texas Southwestern Medical Center, Dallas, TX 75390, USA.; 4 Department of Molecular Genetics, The University of Texas Southwestern Medical Center, Dallas, TX 75390, USA.; 5 Center for Human Nutrition, The University of Texas Southwestern Medical Center, Dallas, TX 75390, USA.; 6 Key Laboratory of Birth Defects and Related Diseases of Women and Children, Department of Paediatrics, West China Second University Hospital, State Key Laboratory of Biotherapy and Collaborative Innovation Center of Biotherapy, Sichuan University, Chengdu 610041, China.; 7 Department of Molecular Biology, The University of Texas Southwestern Medical Center, Dallas, TX 75390, USA.; 8 Division of Hypothalamic Research, Department of Internal Medicine, The University of Texas Southwestern Medical Center, Dallas, TX 75390, USA.; 9 Lead Contact.

## Abstract

Nutrient handling is an essential function of the gastrointestinal tract. Most nutrient absorption occurs in the small intestine and is coordinated by hormone-producing intestinal epithelial cells known as enteroendocrine cells (EECs)^1^. In contrast, the colon mostly reclaims water and electrolytes, and handles the influx of microbially-derived metabolites, including short chain fatty acids (SCFA)^2–4^. Hormonal responses of small intestinal EECs have been extensively studied but much less in known about the role of colonic EECs in metabolic regulation. To address this core question, we investigated a mouse model deficient in colonic EECs. We found that colonic EEC deficiency leads to hyperphagia and obesity. Surprisingly, colonic EEC deficiency results in altered microbiota composition and metabolism, which we found through antibiotic treatment and transfer to germ free recipients, to be both necessary and sufficient for the development of obesity. Moreover, studying stool and blood metabolomes, we found that differential glutamate production by intestinal microbiota corresponds to increase appetite due to EEC loss. Finally, we show that colonic glutamate administration can directly increase food intake and activate appetite centers in the central nervous system. These observations shed light on an unanticipated host-microbiota axis in the colon, part of a larger gut-brain axis, that regulates host metabolism and body weight.

Obesity is a dominant health problem of the 21^st^ century^5^ and is associated with myriad negative health consequences^6^ ranging from diabetes to fatty liver disease, cardiovascular morbidity, and increased risk for a variety of malignancies, which in aggregate, result in excess mortality^7^. In all, obesity can be viewed as the most impactful disease risk factor of our time. The complexity of the biologic processes that control body weight cannot be overstated and include a variety of intricate neuro-hormonal mechanisms. Key among them are hormone producing cells of the intestine, also known as enteroendocrine cells. Their endocrine products coordinate digestion and absorption in the GI tract, as well as metabolic responses in a variety of distant organs, such as the liver, the pancreas, and the hypothalamus. EECs are a diverse population of cells that account for less than 1% of the gastrointestinal epithelium and EECs and remain intensely studied even more than a century^8^. These cells are capable of producing over 20 distinct peptide hormones and monoamine active compounds and are endowed with the ability to sense luminal small molecules as well as circulating metabolites and hormones^9^. Upon the right stimulus, EECs release their hormonal contents, which then exert autocrine, paracrine or endocrine effects, depending on the specific hormone in question.

Initially, EECs were thought to predominantly produce one hormone and were historically subdivided based on their hormone secretion using a letter designation^9^. However, recent single cell RNA-sequencing data identified that most EECs produce several hormones at a time^10,11^, and those hormones are produced by more than one cell type along the GI tract. Moreover, the identity of EECs, as far as their repertoire of hormone expression, undergoes a dynamic switch as cells migrate along the crypt villous axis^12^. Based on their unique gene markers, EECs of the small intestine into 8 distinct groups while 9 subgroups of EECs have been reported in the colon^10,11^. A number of transcription factors are required for epithelial differentiation to EEC^13^, including Neurogenin-3 (*Neurog3)*. Thus, deletion of *Neurog3* along the entire intestinal epithelium of mice (through *Villin*^*Cre*^-mediated recombination), prevents EEC development in both the small and large intestine leading to severe malabsorptive diarrhea, weight loss, and early lethality^14^.

Most attention has been devoted to the metabolic roles of EECs in the proximal GI tract, but the metabolic role of EECs in the colon is much less well understood. Nonetheless, colonic EECs express several hormones known to regulate host metabolism such as GLP-1, GLP-2, INSL5, PYY and NPY^15–18^. Traditionally, the colon has been viewed as an organ mostly responsible for water and electrolyte reabsorption and with limited metabolic roles or contributions. However, it is now clear that through its resident microbiota, the colon is the source of a large flux of small metabolites resulting from fermentation of non-digestible nutrients, which can contribute significantly to host metabolism^19,20^. In fact, it is estimated that bacterial generation of SCFA contributes ~10% of calories in humans^21^. Moreover, the composition and metabolic capabilities of the intestinal microbiota can have a large impact on host metabolism at the level of body weight control^20,22–24^. Hence, mechanisms to sense and respond to the metabolic flux originating in the colon would seem essential to host metabolic homeostasis.

To elucidate the metabolic role of colonic EECs, we generated a mouse model of colonic EEC deficiency by tissue-specific deletion of *Neurog3* in the colon. This was achieved by mating *Neurog3*^*fl/fl*^ animals with *CDX2*^*Cre*^ transgenic mice, resulting in *Neurog3* deletion in the colonic epithelium (cecum to rectum Figure S1A, S1B) effectively deleting all Chromogranin A (CGA) positive EECs in the colon while retaining small bowel EECs, including in the ileum (Figure S1C, D). This model, referred to hereafter as EEC^ΔCol^, displayed normal histology in the GI tract and no appreciable effects on goblet cell populations compared to control animals (littermates without Cre, referred hereafter as wild-type or WT) (Figure S1E). Consistent with the loss of EECs, these mice displayed absent expression of gut hormones selectively in the colon, such as *Gcg*, *Insl5* and *Pyy,* without affecting *Muc2*, a goblet cell gene marker (Figure S2A–D).

Unlike germline hypomorphic mutations of in humans *Neurog3*, which result in malabsorptive diarrhea^25^, or pan-enteric deletion in mice which causes and early lethality^14^, EEC^ΔCol^ mice were born without an early phenotype and lived into adulthood at the same rate that their WT littermates. However, we observed that as these mice aged, they gained significantly more weight than WT littermates (Figure 1A, B). Obesity was due to increase in fat mass, as measured by total body MRI (Figure 1C), and by 6 months of age, EEC^ΔCol^ mice exhibited dysfunctional obesity accompanied by liver steatosis and adipose tissue hypertrophy (Figure 1D). This obesity was enhanced by feeding of high fat diet (HFD), resulting in greater weight gain in EEC^ΔCol^ animals compared to littermate controls (Figure 1E), again due to excess fat mass accumulation (Figure 1F). To identify the main driver of obesity in mutant mice, we performed metabolic cage studies and found that EEC^ΔCol^ animals had significantly higher food intake (Figure 1G), but no changes in energy expenditure, heat production or movement (Figure S3A–C). Next, we examined caloric extraction in the gastrointestinal tract: EEC^ΔCol^ mice had higher stool caloric losses per day (Figure 1H), due to increased stool output without increased caloric density (Figure S3D, E). Colonic EEC deficiency did not result in increased total fatty acid excretion in stool (Figure 1I) or any appreciable alteration in the stool concentration of specific fatty acids (Figure S3F), unlike pan enteric EEC deficiency which results in steatorrhea^14^.

Since colonic EECs produce hormones involved in glucose homeostasis, such as GLP-1, GLP-2, and INSL5, we examined glucose tolerance in EEC^ΔCol^ animals. At 8 weeks of age, prior to the onset of appreciable weight differences between the groups, EEC^ΔCol^ mice displayed higher glycemia than their counterpart controls intraperitoneal glucose administration (Figure S4A), associated with a blunted insulin and GLP-1 response (Figure S4B–D), and normal insulin sensitivity, as assessed by intraperitoneal insulin tolerance testing (Figure S4E–F). HFD-fed EEC^ΔCol^ mice displayed even greater glucose intolerance (Figure S4H), as well as a blunted rise in insulin and GLP-1 in response to glucose (Figure S4I–J). Consistent with the presence of obesity-induced insulin resistance (Figure S4G, L), mutant mice were also hyperinsulinemic at baseline (Figure S4I) and displayed a blunted glycemic drop in response to intraperitoneal insulin administration (Figure S4K, L). Taken together, these findings suggest that colonic EEC deficiency disturbs glucose homeostasis independently of its effects on body weight, possibly secondary to blunted GLP-1 secretion.

Due to occasional germline activity of the *CDX2*^*Cre*^ allele, some of the littermate WT control animals lacking the *CDX2*^*Cre*^ allele had inherited a recombined allele, resulting in mice that were heterozygous for *Neurog3* (referred to as *Neurog3*^*fl/R*^, R standing for recombined). Importantly, *Neurog3*^*fl/R*^ had no appreciable differences from *Neurog3*^*fl/fl*^ mice with regards to weight gain when fed conventional chow or HFD (Figure S5A, B), and no differences in glycemic responses to glucose administration (Figure S5C) indicating that heterozygous global deficiency of *Neurog3* had no evident phenotypes. Nonetheless, to corroborate that the metabolic effects were indeed due to EEC loss and not some extraneous effect, we utilized a post-natal model of deletion. A new conditional allele targeting Exon 2 of *Neurog3* was independently generated (Figure S6A, B), which is distinct from the previously reported *Neurog3*^*fl/fl*^ mice^14^ shown in Figure 1. Animals carrying this conditional allele of *Neurog3* were mated with mice carrying the *CDX2*^*Cre-ERT2*^ allele, expressing a tamoxifen-dependent Cre fusion protein (Figure S6C). Tamoxifen (TMX) administration led to effective post-natal deletion of *Neurog3* expression in the colon (Figure S6D). Moreover, TMX-treated mice developed greater weight gain than vehicle treated animals of the same genotype (Figure S6E) and this was associated with the development of hyperphagia (Figure S6F). To control for possible effects of TMX itself, we performed a repeat experiment where all animals were treated with TMX; Cre-carrying animals experienced excess weight gain compared to non-Cre controls (Figure S6G). Four weeks post-TMX administration, and prior to obesity development, inducible EEC^ΔCol^ animals also demonstrated glucose intolerance (Figure S6H, I). Altogether, post-natal deletion of EECs effectively recapitulated the features of the non-inducible model.

Colonic deletion of *Gcg* (encoding GLP-1, −2, and Glucagon) as well as global deletion of *Insl5* have not been noted to lead to obesity^26,27^. However, whether *Pyy* deletion could itself result in hyperphagia and weight gain in rodents has been debated^28–30^. To test if deficiency of this hormone alone could be responsible for hyperphagia and obesity, we developed a *Pyy* deleted mouse by targeting exons 2 and 3 of the gene (Figure S7A, B). Plasma PYY was essentially undetectable in these mice, as would be expected (Figure S7C). Importantly, these mice did not display excess weight gain on conventional chow or HFD (Figure S7D) and were not hyperphagic compared to littermate controls (Figure S7E). Thus, deficiency of this hormone could not explain the weight phenotype observed in EEC^ΔCol^ animals. To expand our search for other potential hormonal culprits for the obesity phenotype, we examined plasma concentrations of a panel of metabolically relevant hormones. This showed no significant differences between mutant animals and littermate controls, whether in the fasting state or 4 hours post-feeding (Figure S8).

Up until this point, all experiments had been conducted by segregating littermates after weaning according to genotype. This raised the question of whether obesity predisposition in EEC^ΔCol^ mice could be due to extraneous factors, such as microbiome composition^31,32^. To assess this question, we co-housed animals of different genotypes from weaning onwards and placed them on HFD starting at 6 weeks of age. Strikingly, co-housed mutant animals had weight curves undistinguishable from control WT mice; excess weight gain only developed in mutant mice that were genotype-segregated (Figure 2A). Body composition analysis again showed that segregated mutant mice had higher fat mass (Figure 2B). Strikingly, co-housing not only normalized body weight gain under HFD, but also eliminated excess food intake in mutant mice (Figure 2C). Furthermore, obese mutant mice had a clear alteration in their stool microbiota, with decreased microbial diversity (Figure 2D) and skewed composition by β-diversity analysis (Figure 2E). Consistent with this, differential abundance testing utilizing ANCOMBC analysis for amplicon sequence variant (ASV) changes also identified many statistically significant differences between the separately housed EEC^ΔCol^ mice compared to other groups (Figure 2F).

Because obesity itself leads to dysbiosis, we could not conclude whether altered stool microbiota in obese mutant mice was the result of their genotype or was due to their weight differences. To examine this question, we analyzed stool microbiome composition soon after weaning (week 4) and in early pre-obese states (weeks 6–8). In both instances, β-diversity of the stool microbiome of mutant mice had clearly diverged from their littermate WT controls (Figure 2G), at a time that preceded the emergence of weight differences, which were seen only by week 9 (Figure 2H). Interestingly, ANCOMBC also identified that the number of ASV-level changes intensified between 4 and 6–8 weeks, indicating progressive dysbiosis after weaning and separate housing (Figure S9A). Nonetheless, already at week 6, genotype-segregated mice displayed increased food intake compared to control animals (Figure 2I). Finally, we also observed that 5 weeks after TMX-inducible deletion of colonic EECs, when animals had developed hyperphagia but were not yet obese (Figure S6E, F), there was a change in α− and β−diversity, as well as ASV-level composition (Figure S9B). Altogether, these results indicated that dysbiosis and hyperphagia are early events resulting from EEC loss in the colon and that these changes precede obesity.

To elucidate whether microbiota changes in EEC^ΔCol^ animals may be responsible for their obesity phenotype, we utilized broad antibiotics to deplete intestinal microbial populations. Following this, EEC^ΔCol^ mice and littermate controls were placed on HFD, and this time, there was no difference in weight (Figure 3A). Consistent with their weight curves, this treatment also eliminated any differences in food intake between groups (Figure 3B). Pairwise comparisons between antibiotic-treated and vehicle-treated groups indicated that antibiotics did not affect weight gain under HFD in control mice, but greatly blunted weight gain in EEC^ΔCol^ mice (Figure S10A, B), and prevented tissue signs of dysregulated obesity such as hepatic steatosis and adipose hypertrophy (Figure 6C). Thus, obesity and hyperphagia in EEC^ΔCol^ mice is dependent on their intact intestinal microbiota. Next, we sought to establish if the microbiota was sufficient to confer hyperphagia and obesity through transfer experiments into germ-free (GF) wild-type recipient mice. GF recipients of microbiota from iEEC^ΔCol^ mutant mice gained more weight than recipients of microbiota from WT littermate TMX-treated mice; this was evident whether the transplanted mice were on conventional diet (Figure 3D) or HFD (Figure 3F), and in both instances, the weight gain was associated with increased food intake (Figure 3 E, G). As would be expected, the resulting microbiota of GF recipients of WT or mutant donor stool was significantly different at the β-diversity level (Figure 3H), driven by multiple ASV-level differences (Figure 3I).

Next, we hypothesized that microbial-derived metabolites sensed centrally would be ultimately responsible for the hyperphagia observed. Thus, we performed unbiased metabolomic analysis of stool (Figure 4A) contrasting the effects of colonic EEC deletion and controlling for TMX treatment and housing. Specifically, iEEC^ΔCol^ animals housed separately were compared to 4 other control groups. A unique cluster of stool metabolites emerged that was elevated in iEEC^ΔCol^ animals housed separately compared to all 4 control groups (Figure 4A). Next, we performed untargeted metabolomic analysis on blood samples from iEEC^ΔCol^ animals and compared the results with two control groups (Figure 4B). Strikingly, only one metabolite overlapped between blood and stool metabolomics, namely L-glutamic acid, and similarly, blood metabolomic analysis identified that GF recipients of mutant microbiota also displayed increases in blood L-glutamic acid (Figure 4C). Furthermore, compared to 3 control groups, stool glutamate concentrations progressively increased following TMX-mediated deletion of *Neurog3* (Figure 4D), and this phenomenon preceded the onset of hyperphagia and obesity (Figure S6 and S11A and B). Thus, an increase in stool and blood L-glutamic acid correlated with increased appetite and weight gain.

To establish whether this metabolite correlation directly drives increased appetite or is simply associated but not causative, we administered sodium glutamate to WT animals. When provided in the drinking water over a 3-day course, glutamate increased food intake among WT treated animals (Figure 4E). Because oral administration leads to increased luminal glutamate in the small bowel as well as the colon, we examined the effects of rectal administration of glutamate on feeding behavior. Rectal administration of glutamate led to increased food consumption in the ensuing 18 hours (Figure 4F). Next, we examined whether rectal glutamate also activates appetite centers in the brain and found that the number of c-Fos^+^ cells was substantially elevated in the arcuate nucleus of the hypothalamus (ARH) after rectal glutamate administration. Thus, glutamate in the lumen of the large intestine is sufficient to activate central nervous system pathways involved in appetite regulation.

Regarding the source of stool L-glutamic acid, targeted metabolomics indicated that stool L-glutamic acid is essentially undetectable in germ-free mice compared to conventional mice, consistent with the notion that glutamate in stool is largely generated by bacterial metabolism (Figure S11A). Moreover, targeted metabolomics indicated that stool L-glutamic acid concentrations in WT mice dropped upon feeding; in contrast, stool L-glutamic acid concentrations were elevated in iEEC^ΔCol^ mice and did not respond to the feeding cycle (Figure S11B). Finally, obese *ob/ob* (*Lep*^*mut/mut*^) mice display increased stool glutamate compared to WT (*Lep*^*WT/WT*^) non-obese controls (Figure S11E–G). Altogether, we concluded that loss of colonic EECs and the resulting dysbiotic gut microbiota led to higher concentrations of L-glutamic acid in the colon that can lead to increased appetite and obesity.

The development of intestinal microbial dysbiosis in obesity has by now been well documented^33^ and a series of elegant transfer studies into germ-free animals confirmed that microbiota from obese donors can transfer propensity to obesity and metabolic phenotypes to recipient mice, whether the microbiota source is murine or human^31,32,34^. The precise role that this obesogenic activity of microbial dysbiosis plays in weight gain, from inciting to perpetuating factor, remains a point of debate but at least it appears that dysbiosis is not simply incidental. Mechanistically, studies have shown that microbiota from obese hosts is more adept at caloric extraction from food, resulting in a more positive energy balance^32^. Our study identified that EEC loss in the colon can also lead to microbially-mediated metabolic perturbations that promote obesity. Surprisingly, we find that weight gain is driven by alterations in microbial composition and metabolism. Specifically, we identify microbially derived L-glutamic acid production as responsible for activating appetite in the hypothalamus. This conclusion is in agreement with previous studies in humans showing that plasma glutamate was considerably elevated in obese individuals^35^. We also find that *ob/ob* mice have similar stool glutamate elevation, suggesting that this may be a more general feature of obesity in mammals. This concept is further strengthened by prior reports showing that stool transfer from *ob/ob* mice into GF recipients can transfer the obese phenotype^32^, and the observation that antibiotic treatment can temporarily diminish the hyperphagia of the *ob/ob* model^36^. Here we show that stool L-glutamic acid concentrations are negligible in germ-free animals, confirming that bacterial and not host metabolism is mostly responsible for the presence of this amino acid in stool, and also in agreement with metabolomic studies in humans that correlate plasma glutamate with stool microbiota composition^35^. Thus, the relationship between microbial metabolism, glutamate production, appetite regulation and obesity, fits a larger paradigm that extends to human obesity.

As has been noted before^10,26,27,29,37–42^, we also find that key hormones like *Gcg*, *Pyy* and *Insl5* are highly expressed in the distal colon. However, the microbiota and not the hormonal deficiencies seen after colonic EEC ablation, were found to be both necessary and sufficient for hyperphagia and obesity. Indeed, neither *Gcg*^ΔCol^ mice nor global deletion of *Insl5* led to hyperphagia or obesity^26,27^. Nonetheless, some of the metabolic phenotypes seen here likely result directly from specific hormone deficiencies. We observed that EEC^ΔCol^ mice have a blunted 15-minute GLP-1 and Insulin response to glucose challenge, which may be sufficient to explain the glucose intolerance observed in weight-matched mutant mice, akin to the phenotype of *Gcg*^ΔCol^ mice^27^.

This study also establishes that colonic EECs can regulate stool microbiome composition and metabolic activities, uncovering a previously unappreciated enteroendocrine-microbial axis. This relationship between the colonic EEC compartment and the resident microbiota in the colon makes physiologic sense when considering the myriad microbially derived metabolites reaching the host and their overall significant metabolic and caloric contributions^21,43,44^. We postulate that EEC modulation of the microbiota is likely mediated by changes in nutrient availability in the intestinal lumen or by changes in colonic motility, but this remains an area that will require ongoing study. It is also worth mentioning that previous reports have established several effects of the microbiota on EEC function. PYY production and the number of *Pyy*-expressing cells in the colon are stimulated in response to SCFA sensing through FFAR2, a SCFA receptor^41,42^, and Insl5 and Glp-1 production in the colon are regulated by microbial colonization^38,45^. Altogether, our studies and prior reports present an emerging picture in which the colon and its EECs play a critical role in sensing and modulating the influx of microbially derived products to control the metabolic effects on the host. Furthermore, alterations of these processes may play important roles in human obesity.

## METHODS

### Animals:

Animal experiments carried out at UT Southwestern were conducted with approval from its Institutional Animal Care and Use Committee (IACUC) and under supervision of the animal resource center (ARC); animal studies at Chongqing Medical University were performed after approval from its animal ethics committee and following the guidelines of the institutional animal care and use committee at Chongqing Medical University. All mice strains were bred in a C57BL/6J background. Germ-free animals of a C57Bl/6 background were housed in isolators (Class Biologically clean, Ltd) in a facility operated by the commercial vendor Cyagen.

### Mutant mouse strains:

Colonic enteroendocrine deficient animals (EEC^ΔCol^) were generated as previously reported^46^. Briefly, a colonic specific Cre driver mouse^47^, *CDX2*^*Cre*^, was mated against a mouse carrying a *Neurog3* floxed allele^14^. Because of stochastic gonadal recombination events in the *CDX2*^*Cre*^ *Neurog3*^*fl/fl*^ mice, a proportion of the non-Cre offspring carried a single recombined allele at the *Neurog3* locus, which we refer to as *Neurog3*^*R*^. Animals with a single recombined allele (*Neurog3*^*R/fl*^) were experimentally determined to behave as *Neurog3*^*fl/fl*^ (Figure S5); thus, WT controls consisted of both fl/fl and R/fl genotypes.

In later experiments, we utilized tamoxifen (TMX) inducible recombination model, relying on the *CDX2*^*Cre-ERT2*^ transgenic allele^48^. First, a novel conditional mouse model carrying a *Neurog3* floxed allele (Figure S6) was generated through the commercial vendor Cyagen; these mice are distinct from the line derived in the Gradwohl lab and previously reported^14^. Briefly, a *Neurog3* conditional allele was generated by Cyagen utilizing CRISPR/Cas9 endonucleases to replace exon 2 of the murine *Neurog3* gene with a floxed allele. The targeting vector was generated by PCR using the BAC clone RP23–191l4 as a template. These *Neurog3*^*fl/fl*^ mice were mated with animals containing the *CDX2*^*Cre-ERT2*^ transgene and available from Jackson (JAX Strain #035169) ^49^. Traits present in strain #035169, other than *CDX2*^*Cre-ERT2*^ (*Kras*^*G12D*^ and *Apc*^*fl*^), were bred out of the colony to derive *Neurog3*^*fl/fl*^, *CDX2Cre-ERT2* mice.

Leptin deficient mice (*Lep*^*ob*^ *– ob/ob*) were purchased from Germpharmatech. Heterozygote breeding pairs produced litters carrying either one copy of the mutant allele or two copies of the WT (WT) or mutant alleles (*ob/ob*). For experiments, only WT or *ob/ob* mice were used.

Knockout mice for *Pyy* were generated by the commercial vendor Cyagen through CRISPR/Cas9 endonuclease recombination that resulted in a ~506bp deletion of the *Pyy* locus that includes deletion of exons 2 and 3. Breeding pairs carrying a single deleted *Pyy* allele produced offspring at expected Mendelian rates, born with either a single copy of the mutant allele (*Pyy* ^+/−^), two copies of the WT allele (*Pyy* ^+/+^), or two copies of the deleted allele (*Pyy* ^−/−^). For experiments, only *Pyy* ^+/+^ (WT) or *Pyy* ^−/−^ mice were used.

### General husbandry, diets, antibiotics and tamoxifen treatments:

Other than germ-free experiments, mouse strains were kept in a specific pathogen-free (SPF) facility with a standard 12-hour light-dark cycle and free access to chow. In all experiments, except cohousing experiments, mice were segregated by sex and genotype at time of weaning. Regular chow consisted of 3 kcal/g (Teklad global 2016/2916), whereas high fat diet consisted of 60% fat and 5.24kcal/g (Research Diets D12492). Antibiotic treatments consisted of drinking water supplemented with Neomycin (1g/L), Metronidazole (1g/L), Ampicillin (1g/L) and Vancomycin (500mg/L). Tamoxifen (Sigma Aldrich) was diluted in corn oil (Selleck) to a concentration of 10mg/ml. A total dose of 2mg was administered daily by oral gavage to 8-week-old age mice over a 3-day period.

### Fecal microbiota transplantation:

Fresh morning fecal pellets were collected from 5, TMX-treated, single housed *Neurog3*^fl/fl^ or 5 iEEC^ΔCol^ mice, 5 weeks after TMX administration (at 13 weeks of life). The stool was snap frozen in liquid nitrogen and stored at −80°C until use. On the day of transfer, stool was thawed, and under sterile conditions, pellets from corresponding donors were mixed. A sterile fecal suspension was prepared in 0.9% NaCl at 10% w/v. The solution was thoroughly mixed and later filtered to remove large particles. Finally, the suspension was centrifuged, and the supernatant collected for transfer. A total volume of 200μl/dose of the fecal suspension was transferred to 8-week-old germ-free animals on 6 occasions via oral gavage over a 2-week period. Recipient mice were weighed weekly. Recipient animals on conventional diets remained in isolators for the remainder of the experiment, while those placed on HFD were transferred to a SPF facility upon transfer completion. Stool for 16s analysis (see below) was collected from recipient mice at 12 weeks of age.

### Glutamate treatments:

For oral treatment experiments, individually housed adult (average age 16 weeks) male *Neurog3*^R/fl^ animals were conditioned with autoclaved water via bottle for 4 days prior to obtaining baseline body weight. Afterwards, mice were provided either a 5% (w/v) sodium glutamate prepared in autoclaved water) or water for a period of 3 days via a drinking bottle. Chow weight was measured and recorded daily. For rectal glutamate administration, a 5% (w/v) sodium glutamate enema was prepared by mixing glutamate and a gel solution comprised of 8% w/v food thickener (modified food starch and maltodextrin) and water. Control gel was prepared without glutamic acid. Single-housed 9-week-old WT male animals were anesthetized in the early evening with inhaled isoflurane. A clearing enema of PBS was administered, and mice were allowed to roam freely in their cage for 60 min. Mice were then re-anesthetized and given a 500μl glutamate or control gel enema. Immediately afterwards, a dark cycle was begun, and chow was weighed at the beginning of the experiment and 18h afterwards.

### Metabolic cages:

Metabolic cage experiments were carried out by the University of Texas Southwestern’s Metabolic Phenotyping Core facility. Adult male mice of either genotype (EEC^ΔCol^ or littermate wild-type controls lacking the *CDX2*^*Cre*^ transgene) were individually housed in metabolic chambers maintained in a 12-hour light-dark cycle. Continuous metabolic parameters (O_2_ consumption, CO_2_ production, locomotion, and food/drink consumption at room temperature) were recorded using the TSE PhenoMaster indirect calorimetry system after a 5-day acclimatization period.

### MRI:

Total fat mass was measured by Eco-MRI from University of Texas Southwestern’s Metabolic Phenotyping Core facility.

### Co-housing:

Littermate male mice (EEC^ΔCol^ or WT controls) were raised to experimental age (as noted in figures) together (co-housed) as siblings to avoid in-fighting. All cohoused animals were visually inspected for fight wounds and only mice who were harmoniously paired were included in experiments.

### Non-metabolic cage food consumption:

Food consumption was determined by daily weighing chow pellets in the containing cage. Each cage housed an individual animal. We standardized consumption to daily mouse weight and calculated an average over a 7-day period.

### Stool fat LC/MS:

Stool from individually housed HFD-fed male (started at 8 weeks of age) mice, was obtained at 16 and 23 weeks of life, after 48h collection period in a wire bottom cage. The stool was carefully inspected to be free of fur or other potential contaminants. After a 48h period, the stool was pulverized and submitted for LC/MS. Approximately 50mg of mouse stool was homogenized in methanol/dichloromethane (1:2, v/v) using a Bead Ruptor. The homogenates were further diluted to a final concentration of 20 mg/mL using methanol/dichloromethane (1:2, v/v). Total fatty acid profiles were generated by a modified GC-MS previously described method^50^. Briefly, lipids were extracted by adding 1mL each of dichloromethane, methanol, and water to a glass tube containing the sample. The mixture was vortexed and centrifuged resulting in two distinct liquid phases. The organic phase (lower phase) was placed in a fresh glass tube with a Pasteur pipette and dried under N_2_. The dried extracts were resuspended in 1mL of 0.5M potassium hydroxide solution prepared in methanol, spiked with 100μL of 0.5μg/mL of fatty acid standards (FA(16:0[2H31]), FA(20:4ω6[2H8]) and FA(22:6ω3[2H5]), and hydrolyzed at 80°C during 60 minutes. Hydrolyzed fatty acids were extracted by adding 1mL each of dichloromethane and water to the sample in hydrolysis solution. The mixture was vortexed and centrifuged for 5 minutes, and the organic phase was collected to a fresh glass tube and dried under N_2_. Dried extracts were resuspended in 50μL of 1% triethylamine in acetone, and derivatized with 50μL of 1% pentafluorobenzyl bromide (PFBBr) in acetone at room temperature for 25 min in capped glass tubes. Solvents were dried under N_2_, and samples were resuspended in 500μL of isooctane. Samples were analyzed using an Agilent 7890/5975C (Santa Clara, CA, USA) by electron capture negative ionization (ECNI) equipped with a DB-5MS column (40m × 0.180mm with 0.18μm film thickness) from Agilent. Hydrogen (carrier gas) flow rate was 1.6mL/min and injection port temperature were set at 300°C. Sample injection volume was 1μL. Initial oven temperature was set at 150°C, and then increased to 200°C at a 25°C/min, followed by an increase of 8°C/min until a temperature of 300°C was reached and held for 2.2 minutes, for a total run time was 16.7 minutes. Fatty acids were analyzed in selected ion monitoring (SIM) mode. The FA data was normalized to the internal standards. Fatty acid with carbon length; C ≤ 18 were normalized to FA(16:0[2H31]), C = 20 were normalized to FA(20:4 ω6[2H8]), and C = 22 were normalized to FA(22:6 ω3[2H5]). Data was processed using MassHunter software (Agilent).

### Stool bomb calorimetry:

Stool was obtained from individually-housed male animals of either genotype (EEC^ΔCol^ or WT – *Neurog3*^fl/fl^ or *Neurog3*^R/fl^) fed regular chow. Mice were placed in wire bottom cages for a 48h period, and the entire stool output collected from the cage. Stool was submitted to the UTSW Mouse Metabolic Phenotyping Core, which uses a Parr 6200 Isoperibol Calorimeter Equipped with a 6510 Parr Water Handling system for calorific tests. Gross heat of sample combustion is reported and normalized per total stool output in a 24h period.

### Tissue staining:

Tissue immunofluorescence was carried out as previously reported ^46^. Briefly, after CO_2_ euthanasia, liver, bowel and abdominal fat were carefully dissected, rinsed with PBS and fixed overnight in 4% paraformaldehyde containing PBS (Sigma Aldrich). For immunofluorescence of intestinal tissues, paraffin blocks were prepared from PFA-fixed tissue in standard manner and 4 μm microtome-sectioned scrolls used for mounting onto slides. We performed slide deparaffinization through xylene, heating and serial ethanol rehydration. Tissues were stained with hematoxylin and eosin (HE) or alcian blue using standard protocols. For immunofluorescence staining, antigen retrieval was performed using a warm citrate solution. Thereafter, slides were blocked using 3.5% goat serum diluted in PBS for 60min. Primary antibody (rabbit-anti CHGA – ab15160) diluted in blocking buffer was then applied at a dilution of 1:150. Slides were incubated overnight at 4°C in primary antibody. After washing in PBS, slides were then incubated for 60min in a fluorescent secondary antibody (goat-anti mouse Alexa555). Slides were washed in PBS and nucleus was stained using Hoechst 33342 (Sigma Aldrich) diluted at 1:10,000 in PBS for 20 min. After a final washing step in PBS, slides were mounted with SlowFade Gold Antifade Mountant (Invitrogen). Fluorescent images were captured using a Nikon A1R confocal system and a Keyence BZ-X800 fluorescence microscope. Light microscopy images were captured using the Keyence system.

### cFos staining and quantification:

Mouse brains were prepared as previously described^51^. Anti-cFos (Abcam, US; Catalog# ab190289, Lot#CR3418522–1, RRID:AB_2737414) and secondary fluorescent antibodies (Thermofisher Inc, US; Catalog# A32790, Lot#WI320931, and RRID:AB_2762833) were used. Dilution rates for antibodies were 1:1000 for the first antibody and 1:200 for the second antibodies. Images were captured by fluorescence microscope (Leica Inc, US; Model DM6 B). Exposure of captured images was adjusted, and each the arcuate hypothalamic nucleus was clipped by Photoshop. Clipped images were exported to Fiji, and the number of cells expressing Fos was counted by particle measurement function.

### Glucose and insulin tolerance studies:

For intraperitoneal glucose tolerance test (IPGTT), genotype-segregated male mice who had been morning-fasted (4h) received glucose by intraperitoneal injection (2g/kg). Tail vein glycemia was measured using a glucometer (Bayer Contour Blood Glucose Meter, Model 7189) and 30μL of blood was collected into DDP4 primed vessels at the time points indicated in the figures. Glp-1 and insulin concentration were determined by ELISA (Crystal Chem). For Insulin tolerance tests (ITT), 4-h fasted genotype-segregated male mice were intraperitoneally injected with a dose of 0.5U insulin per kg body weight, and tail vein glycemia was measured using a glucometer at the indicated time points.

### Hormone measurements:

For PYY measurements, tail blood was collected from genotype-segregated 8-week old male animals after an overnight fast. Serum PYY concentration was determined by ELISA (FUJIFILM Wako). A milliplex (Millipore) assay was used to determine serum concentrations of 9 different hormones. Blood was collected from 11-week-old genotype-segregated male mice after an 18h overnight fast and 4h after refeeding.

### Tissue gene expression:

Adult mice were euthanized by CO_2_ asphyxia. Immediately afterwards the entire colon and small bowel were harvested, and intestinal contents rinsed with ice-cold PBS. The bowel was then sectioned into 6 pieces (duodenum, jejunum, ileum, cecum, proximal and distal colon), placed in RNAlater (QIAGEN) at 4°C overnight and then stored at −80°C. RNA extraction was performed from RNAlater-stabilized tissue using Qiagen’s RNeasy mini spin columns according to the manufacturer’s instructions. RNA was reverse transcribed using the SuperscriptIII system (Invitrogen) following the manufacturer’s instructions. Real-time PCR was performed using the intercalated dye SYBR green system (Applied Biosystems) utilizing mouse specific primers for genes of interest. Gene expression quantification was performed using the delta-delta Ct method.

### 16s sequencing:

Fecal microbiota analysis by 16s sequencing was performed as previously reported^46^. Freshly-defecated morning stool pellets were collected from mice and snap-frozen in liquid nitrogen. Stool DNA was extracted using either the Powerfecal system (QIAGEN) or Magnetic soil and stool DNA kit (Tiangen) as per instructions from the manufacturer. Sequencing of the V4 region of the 16sRNA gene was carried out at UTSW microbiome sequencing core facility using a MiSeqDx instrument, or by Metware Biotechnology Co., Ltd using a NovaSeq 6000 instrument. The QIIME2 v.2022.8 pipeline was used for FASTQ sequence analysis^52^. Paired-end sequences were initially demultiplexed, trimmed and filtered. After phylogenetic profiling, alpha and beta diversity were analyzed. Sequences were aligned and taxonomically assigned using the SILVA database release 138. Differential abundance testing was performed using the ANCOMBC pipeline^53^. ASV’s were determined to be differentially abundant by FDR corrected p<0.05.

### Metabolomic analysis:

**Figure 4:**
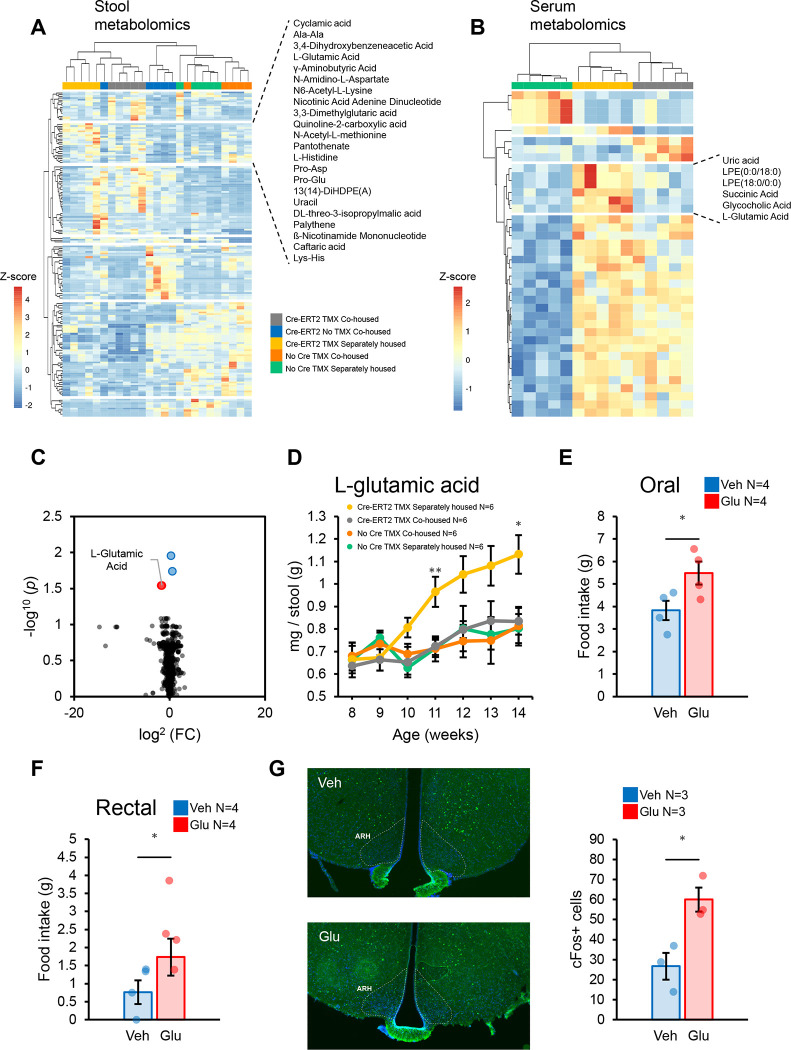
Microbially-derived glutamate drives hyperphagia. **(A)** Heatmap with hierarchical clustering of statistically significant FDR-corrected stool metabolites after ANOVA testing in the animal groups depicted. Stool metabolites significantly enriched in the genotype segregated iEEC^ΔCol^ group are highlighted. **(B)** Heatmap with hierarchical clustering of statistically significant FDR corrected serum metabolites after ANOVA testing in the animal groups depicted. Serum metabolites significantly enriched in the genotype segregated iEEC^ΔCol^ group are highlighted. **(C)** Volcano plot of serum metabolites from 12-week-old GF animals after FMT of stool from iEEC^ΔCol^ or TMX treated WT mice. FDR-corrected statistically significant (by unpaired t-test) metabolites are color highlighted and labeled. **(D)** Time course of fecal L-glutamic acid following TMX treatment at 8 weeks of age in the mouse groups depicted; measurements were made after 4h fast. **(E)** Average food intake of individually housed adult WT mice after 3-day on water supplemented with sodium glutamate or water control (average age 16 weeks). **(F)** Overnight food intake of individually housed 9-week-old WT mice after rectal sodium glutamate administration via enema. **(G)** Quantification of cFos+ cells in the hypothalamus at Bregma −2.06, determined 6 hours after rectal sodium glutamate administration. Representative images (left) and quantification in individual mice (right) are shown. Mean values are represented by bars and individual values by jitter plots. Error bars are ± S.E.M. Two-tailed unpaired t-test used for all pairwise comparisons. *p<0.05.

After a 4h fast, morning serum and stool was collected from 12-week old mice from groups indicated in Figure 4 A and B. The samples were frozen and stored at −80°C. Targeted and untargeted metabolic profiling was performed by Metware Biotechnology. Statistical analysis of metabolomics data^54^ was performed using metaboanalyst v5.0. Single factor analysis was used with QC filtering at 25%. Data was mean-normalized and log-transformed. Statistical analyses with ANOVA or unpaired t-test were used depending on group number. All significant features were FDR corrected and an alpha of 0.05 was considered significant.

### Statistics:

Statistical testing of pairwise comparisons for quantitative data was performed using unpaired 2-tailed t-test as detailed in individual figured legends. We utilized excel and the XLSTAT 2019 add-in for excel to perform these analyses. For 16s sequencing data, alpha diversity pairwise comparisons between groups, a Kruskal Wallis test was used. In the case of beta diversity, we performed unweighed UNIFRAC and analysis of pairwise comparisons between groups was performed using PERMANOVA. The QIIME2 software was used for 16s analysis, along with customized pipelines that employed R (release 4.0.3) for differential abundance testing as detailed above.

## Supplementary Material

Supplement 1

## Figures and Tables

**Figure 1:**
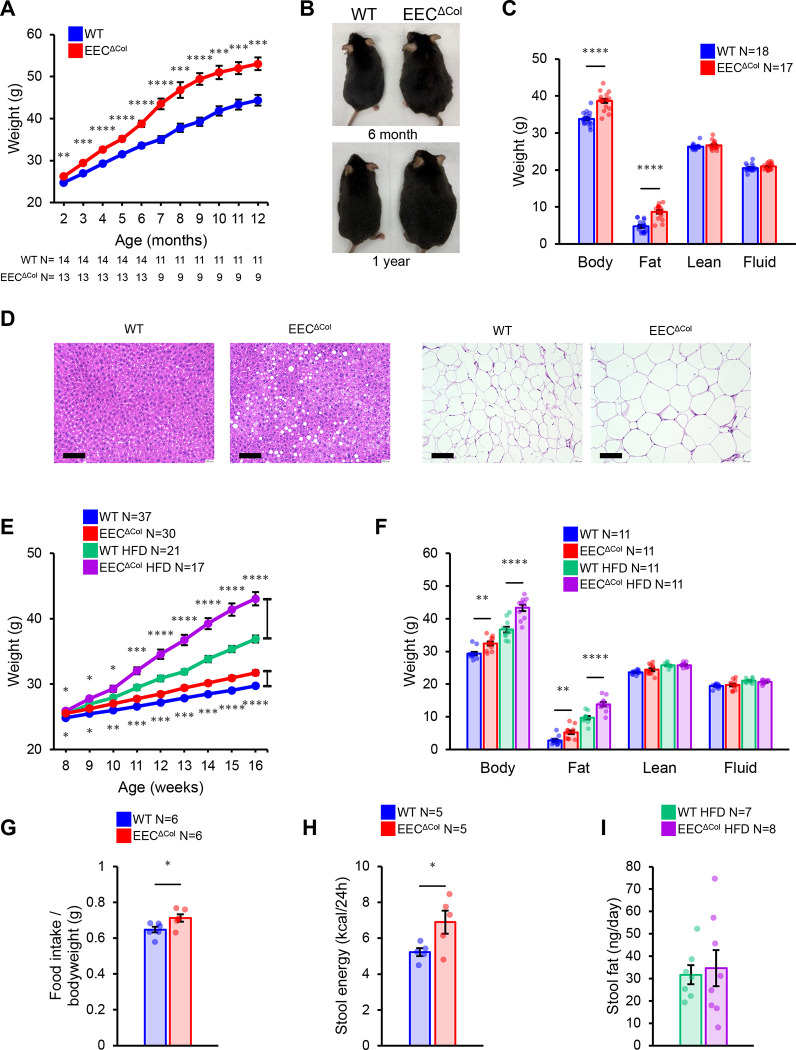
Loss of colonic EECs results in hyperphagia and obesity. **(A)** Body weight curve of EEC^ΔCol^ or WT regular chow-fed male mice. **(B)** Representative images of mice at 6 (top) and 12 months (bottom). **(C)** Body composition at 6-months as measured by total body MRI. **(D)** Representative microphotographs of HE-stained liver (left panels) and adipose tissue (right panels), scale bar 100μm. **(E)** Weight curve for EEC^ΔCol^ or WT HFD-fed and regular chow-fed male mice and **(F)** corresponding body composition as measured by total body MRI at 16 weeks. **(G)** Daily food intake from 10-week-old male mice. **(H)** Stool bomb calorimetry from 11-week-old male mice on conventional diet. **(I)** Stool fat output from HFD-fed male mice (16 and 23-week-old mice were included). Mean values are represented by bars and individual values by jitter plots. Error bars are ± S.E.M. Two-tailed unpaired t-test used for all pairwise comparisons. *p<0.05, **p<0.01, ***p<0.001, ****p<0.0001.

**Figure 2:**
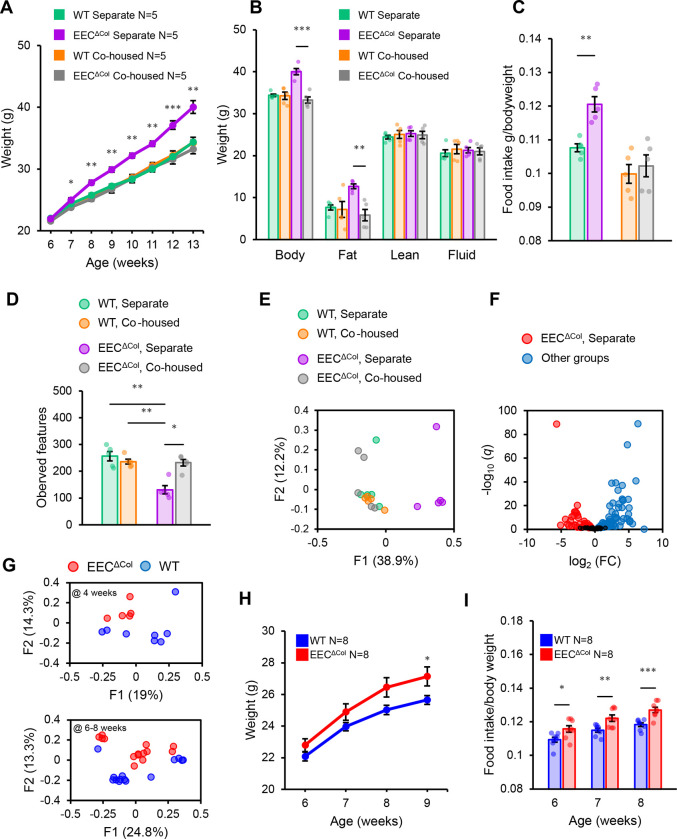
Colonic EEC loss causes intestinal dysbiosis and is associated with obesity. **(A)** Weight curve, **(B)** body composition determined by MRI (at 13 weeks), and **(C)** daily food intake (at 13 weeks of age) of regular and HFD-fed mice who were either co-housed or genotype segregated. **(D)** Alpha diversity by mean ASV richness of fecal 16s rRNA sequencing of stool from animals in (A). **(E)** Beta diversity by unweighed UNIFRAC PCoA of fecal 16s rRNA sequencing of stool from animals in (A), pairwise comparison of EEC^ΔCol^ separately housed animals against all other groups. PERMANOVA p. 0.001 q. 0.001. **(F)** Volcano plots of differential abundance testing by ANCOMBC of fecal microbiota species (sequence variants) from samples in (A). FDR corrected statistically significant species are color highlighted. **(G)** Beta diversity principal coordinate analysis (PCoA) plot determined by unweighed UNIFRAC on stool 16s sequencing from genotype segregated regular chow fed male animals at 4 weeks of life (top panel – PERMANOVA p. 0.01 q0.01) or 6–8 weeks of life (bottom panel – PERMANOVA p.0.002 q.0.002). **(H)** Daily food consumption and **(I)** weekly weight gain of 6- to 9-week-old male mice on regular-chow. Mean values are represented by bars and individual values by jitter plots. Error bars are ± S.E.M. Two-tailed unpaired t-test used for all pairwise comparisons. *p<0.05, **p<0.01, ***p<0.001.

**Figure 3:**
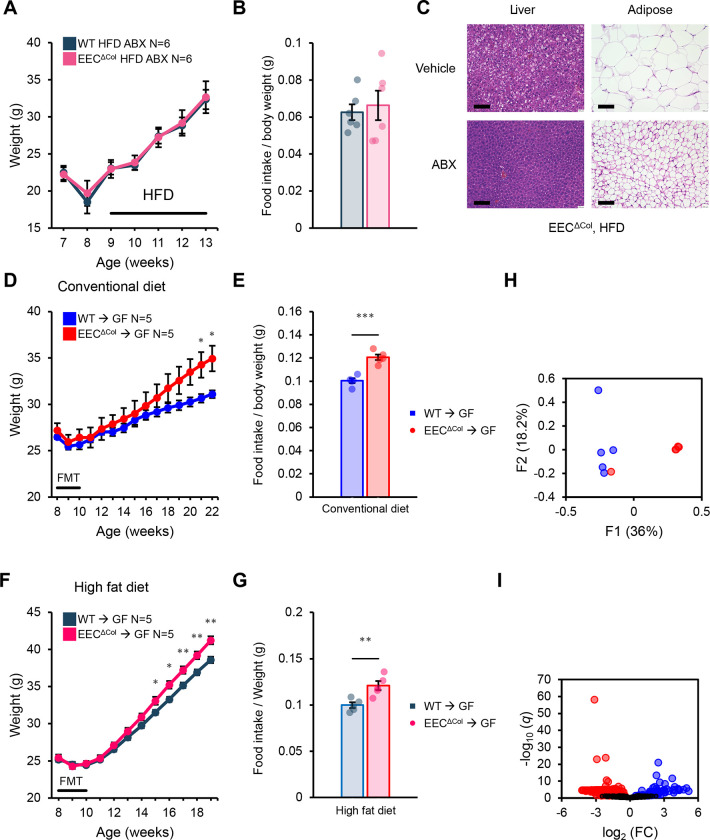
Dysbiosis in EEC^ΔCol^ mice leads to hyperphagia and obesity. **(A)** Weight curves of antibiotic-treated (ampicillin, neomycin, vancomycin and metronidazole) mice of the indicated genotypes on HFD. **(B)** Average food intake of mice in (A) at 13 weeks of life. **(C)** Representative microphotographs of HE stained liver (left panels) and fat (right panels) of vehicle (Veh) or antibiotic (ampicillin, neomycin, vancomycin and metronidazole) treated EEC^ΔCol^ HFD-fed mice. **(D)** Weight curves (on conventional diet) of WT germ-free animals after FMT of stool from iEEC^ΔCol^ or TMX treated control mice. **(E)** Average food intake of mice in (D) at 18 weeks of life. **(F)** Weight curves (on HFD) of WT germ-free animals after FMT of stool from iEEC^ΔCol^ or TMX treated control mice. **(G)** Average food intake of mice in (F) at 14-weeks of life. **(H)** Beta diversity principal coordinate analysis (PCoA) plot determined by unweighed UNIFRAC on stool 16s sequencing from animals in (D). PERMANOVA p. 0.024 q. 0.024. **(I)** Volcano plots of differential abundance testing of fecal microbiota species (sequence variants) from samples in (H). FDR-corrected statistically significant species are color highlighted. Mean values are represented by bars and individual values by jitter plots. Error bars are ± S.E.M. Two-tailed unpaired t-test used for all pairwise comparisons. *p<0.05, **p<0.01, ***p<0.001.

**KEY REAGENTS:** 

REAGENT OR RESOURCE	SOURCE	IDENTIFIER
***Antibodies***		
Anti CHGA	Abcam	Cat# ab15160; RRID:AB_301704
Alexa555	Invitrogen	Cat# A-21428; RRID:AB_2535849
***Chemicals***		
Vancomycin	Sigma Aldrich	Cat# 1404-93-9
Neomycin	Sigma Aldrich	Cat# 1405-10-3
Ampicillin	Sigma Aldrich	Cat# A9518
Metronidazole	Sigma Aldrich	Cat# M3761
Clindamycin	Sigma Aldrich	Cat# 21462-39-5
Tamoxifen	Sigma Aldrich	Cat# 10540-29-1
Corn oil	Selleck	Cat# 8001-30-7
SlowFade Gold anti-fade Reagent	Invitrogen	Cat# S36937
Citrate buffer	Sigma Aldrich	Cat# C9999-1000ML
Glucose	Sigma Aldrich	Cat# G8270
L-glutamic acid monosodium salt monohydrate	Sigma Aldrich	Cat# 49621-250G
Hoechst 33342	Sigma Aldrich	Cat# 94403-1 ML
Insulin	Eli Lilly	Humulin R
Paraformaldehyde	Sigma Aldrich	Cat# P6148-1KG
Rodent Diet With 60 kcal% Fat	Research Diets	Cat# D12492
RNAlater	Qiagen	Cat# 76106
SYBR green	Applied Biosystems	Cat# 4367662
***Experimental models: Organisms/Strains***		
Mouse: C57Bl/6J	Jackson	Cat# 000664
Mouse: CDX2P-NLS Cre; B6.Cg-Tg (CDX2-cre) 101 Erf/J	Jackson	Cat# 009350
Mouse: B6.Cg-Krastm4Tyj Apctm1Tno Tg(CDX2-cre/ERT2)752Erf/MaraJ	Jackson	Cat# 035169
Mouse: Ngn3^flox/flox^; Neurog3^tm3.1Ggr^	Donated to Burstein lab by Leiter and Gradwohl	As published^14^
Mouse: B6/JGpt-Lepem1Cd25/Gpt	GemPharmatech	Cat# T001461
Mouse: C57BL/6J-Neurog3em1cyagen	Cyagen	Custom made for this paper
Mouse: C57BL/6J-PYYcyagen	Cyagen	Custom made for this paper
***Oligonucleotides***		
Neurog3 Flox FWD TCTCGCCTCTTCTGGCTTTC	Sigma Aldrich	Custom made
Neurog3 Flox REV CGGCAGATTTGAATGAGGGC	Sigma Aldrich	Custom made
Neurog3 Rec AACTCCAAAGGGTGGATGAGGGGCG	Sigma Aldrich	Custom made
Neurog3 F1 ACTGGTGTTCTCAGACTTCTTGTG	Sigma Aldrich	Custom made
Neurog3 R1 CTAGGGATCCAAGTAGTGAAAGCC	Sigma Aldrich	Custom made
Neurog3 F2 GGGAGAACTAGGTAACAATTCGGA	Sigma Aldrich	Custom made
Neurog3 R2 GGGAAAAGGTTGTTGTGTCTCTGG	Sigma Aldrich	Custom made
PYY F1 CGCAGCTTTCTTCCCTCATC	Sigma Aldrich	Custom made
PYY R1 CATGCAAGTGAAGTCGGTGT	Sigma Aldrich	Custom made
Cre FWD GCACGTTCACCGGCATCAAC	Sigma Aldrich	Custom made
Cre REV CGATGCAACGAGTGATGAGGTTC	Sigma Aldrich	Custom made
Cre ERT2 FWD TACCGGAGATCATGCAAGC	Sigma Aldrich	Custom made
Cre ERT2 REV GGCCAGGCTGTTCTTCTTAGA	Sigma Aldrich	Custom made
Neurog3 qPCR FWD CCAAGAGCGAGTTGGCACT	Sigma Aldrich	Custom made
Neurog3 qPCR REV CGGGCCATAGAAGCTGTGG	Sigma Aldrich	Custom made
Gcg qPCR FWD TTACTTTGTGGCTGGATTGCTT	Sigma Aldrich	Custom made
Gcg qPCR REV AGTGGCGTTTGTCTTCATTCA	Sigma Aldrich	Custom made
Insl5 qPCR FWD CCCCACTCTTGCTCTGTTTCT	Sigma Aldrich	Custom made
Insl5 qPCR REV GGAAATGCCCCTCCAGATGTC	Sigma Aldrich	Custom made
Pyy qPCR FWD ACGGTCGCAATGCTGCTAAT	Sigma Aldrich	Custom made
Pyy qPCR REV GACATCTCTTTTT CCATACCGCT	Sigma Aldrich	Custom made
Muc2 qPCR FWD GCCTGTTT GATAGCTGCTATGTGCC	Sigma Aldrich	Custom made
Muc2 qPCR REV GTTCCGCCAGTCAATGCAGACAC	Sigma Aldrich	Custom made
***Commercial Kits***		
Powerfecal DNA Kit	Qiagen	Cat# 51804
Mouse GLP-1 ELISA Kit	Cystal Chem	Cat# 81508
Ultra Sensitive Mouse Insulin ELISA Kit	Cystal Chem	Cat# 90080
Mouse/Rat PYY ELISA Kit Wako	FUJIFILM Wako	291-73501
MILLIPLEX^®^ Mouse Metabolic Hormone Expanded Panel	Millipore	MMHE-44K
SuperscriptIII	Invitrogen	Cat# 18080044
RNeasy mini	Qiagen	Cat# 74104
***Software***		
XLSTAT	Addinsoft 2019	https://www.xlstat.com XLSTAT, RRID:SCR_016299
QIIME2		https://qiime2.org QIIME2, RRID:SCR_021258
Metaboanalyst 5.0		https://www.metaboanalyst.ca/ MetaboAnalyst, RRID:SCR_015539
ANCOMBC		https://github.com/FrederickHuangLin/ANCOMBC
FiJi	National Institutes of Health	https://imagej.net/software/fiji/ Fiji, RRID:SCR_002285
Biorender		https://biorender.com/ Biorender, RRID:SCR_018361

## INCLUSION AND DIVERSITY

The authors are primarily composed of scientists from diverse ethnic backgrounds, including underrepresented minorities in fields of science (AL, TFW, LS-D, EB).
