# Supplementary material for: An enteroendocrine-microbial axis in the large intestine controls host metabolism
Source: Res Sq. 2023 Jul 6:rs.3.rs-3112286. Preprint. [Version 1] doi: 10.21203/rs.3.rs-3112286/v1 (PMC10350199; doi:10.21203/rs.3.rs-3112286/v1)
Supplement: Supplement 1 [file NIHPPrs3112286v1-supplement-1.pdf]

SUPPLEMENTAL INFORMATION

Supplementary Figure 1: Generation and validation of EEC deficient mice. **(A)** Schematic representation of the colonic EEC deficiency model. **(B)** qRT-PCR of *Neurog3* gene along regions of the murine bowel **(C)** Morphometric quantification of chromogranin A (CGA) labeled cells along the intestinal epithelium. At least 10 confocal-acquired immunofluorescence images from each intestinal segment (duodenum, jejunum, ileum, cecum, proximal colon, distal colon) from 3 adult mice of either genotype were used for morphometric analysis. Chromogranin A (CHGA) positive cells were visually counted and normalized to the number of cells per 10 intestinal crypts. **(D)** Representative confocal images of immunofluorescent stained regions of the mouse intestine. **(E)** Representative light microscopy images of HE and Alcian blue stained regions of the murine intestine. Mean expression values are represented by bars and individual values by jitter plots. Error bars are ± S.E.M. Two-tailed unpaired t-test used for all pairwise comparisons. $^{**}p<0.01$, $^{***}p<0.001$, $^{****}p<0.0001$.

Supplementary Figure 2: Absent hormone expression in colonic tissue of EEC deficient mice. **(A-C)** qRT-PCR of gut hormone genes along regions of the murine bowel. **(D)** qRT-PCR of *Muc2* (a goblet cell related marker) along regions of the murine bowel. Mean expression values are represented by bars and individual values by jitter plots. Error bars are ± S.E.M. Two-tailed unpaired t-test used for all pairwise comparisons. $^{*}p<0.05$, $^{**}p<0.01$, $^{***}p<0.001$.

Supplementary Figure 3: Metabolic cage analysis of $EEC^{\Delta Col}$ and WT control. **(A)** Heat production, **(B)** energy expenditure and **(C)** total movement was examined in 10-week-old WT or $EEC^{\Delta Col}$ mice. **(D)** Daily stool output and **(E)** stool bomb calorimetry from 11-week-old male WT and $EEC^{\Delta Col}$ mice. **(F)** Concentration of individual fatty acids from stool of 16 and 23-week-old HFD-fed male $EEC^{\Delta Col}$ or WT (aggregate of two repeats) mice. Mean values are represented by bars and individual values by jitter plots. Error bars are ± S.E.M. Two-tailed unpaired t-test used for all pairwise comparisons. $^{*}p<0.05$.

Supplementary Figure 4: Glucose tolerance analysis of $EEC^{\Delta Col}$ and WT control. **(A)** Weight of experimental mice at time of IP glucose administration. **(B)** Intra-peritoneal glucose tolerance test (IPGTT). **(C)** Serum Insulin and **(D)** serum GLP-1 determination following IP glucose load on 8-week-old regular chow-fed male mice. **(E)** Intraperitoneal insulin tolerance test and **(F)** weight of 8-week-old regular chow fed male mice. **(G)** Weight of experimental mice at time of IP glucose administration. **(H)** IPGTT, **(I)** serum insulin and **(J)** serum GLP-1 determination following IP glucose load on 8-week-old HFD-fed male mice. **(K)** Intraperitoneal insulin tolerance test and **(L)** weight of 8-week-old HFD-fed male mice. Mean values are represented by bars and individual values by jitter plots. Error bars are ± S.E.M. Two-tailed unpaired t-test used for all pairwise comparisons. $^{*}p<0.05$, $^{**}p<0.01$, $^{***}p<0.001$, $^{****}p<0.0001$.

Supplementary Figure 5: Neurog3 heterozygous animals do not display metabolic phenotypes. **(A)** Body weight curve of $Neurog3^{fl/fl}$ or $Neurog3^{R/fl}$ regular chow-fed male mice. **(B)** Weight curve for 8-week-old $Neurog3^{fl/fl}$ or $Neurog3^{R/fl}$ HFD-fed and regular chow-fed male mice. **(C)** Weight and Intra-peritoneal glucose tolerance test (IPGTT).

Supplementary Figure 6: An inducible model of colonic EEC loss recapitulates the hyperphagia induced obesity phenotype. **(A)** Targeting strategy for generation of novel *Neurog3* conditional floxed allele. **(B)** Representative genotyping gels of novel *Neurog3* floxed allele. **(C)** Schematic representation of the inducible colonic EEC deficiency model. **(D)** qRT-PCR of *Neurog3* along regions of the murine bowel. **(E)** Weight curve and **(F)** food intake for vehicle (Veh) or tamoxifen (TMX) treated $iEEC^{\Delta Col}$ male mice on a regular diet. **(G)** Weight curve for vehicle tamoxifen (TMX) treated $iEEC^{\Delta Col}$ or non-Cre control mice on a regular diet. **(H)** Weight and **(I)** Intra-peritoneal

glucose tolerance test (IPGTT) for 12-week-old vehicle (Veh) or tamoxifen (TMX) treated iEEC$^{\Delta Col}$ male mice on a regular diet. Mean values are represented by bars and individual values by jitter plots. Error bars are ± S.E.M. Two-tailed unpaired t-test used for all pairwise comparisons. *p<0.05, **p<0.01, ***p<0.001.

Supplementary Figure 7: *Pyy* deficiency model. **(A)** Targeting strategy for generation of a *Pyy* knockout allele. **(B)** Representative genotyping gels of novel *Pyy* knockout allele. **(C)** PYY plasma levels after overnight fast from serum of 8-week-old genotype-segregated male mice of either genotype on regular chow. **(D)** Weight curve for *Pyy* $^{-/-}$ or *Pyy* $^{+/+}$ HFD-fed and regular chow-fed male mice and **(E)** average daily food intake at 8 weeks of age. Mean values are represented by bars and individual values by jitter plots. Error bars are ± S.E.M. Two-tailed unpaired t-test used for all pairwise comparisons. ****p<0.0001.

Supplementary Figure 8: Circulating plasma levels of metabolic hormones. **(A-I)** Milliplex ELISA for respective hormones from serum of 10 and 19-week-old genotype-segregated male mice, after a 18h fasting state, or upon (4h) refeeding. Mean values are represented by bars and individual values by jitter plots. Error bars are ± S.E.M. Two-tailed unpaired t-test used for all pairwise comparisons. **p<0.01.

Supplementary Figure 9: Microbiome analysis of EEC deficiency model. **(A)** Volcano plots of differential abundance testing of fecal microbiota species (ASV) from EEC$^{\Delta Col}$ or WT mice at 4 weeks (left panel) or 6-8 weeks of age (right panel). FDR-corrected statistically significant species are color highlighted. **(B)** Alpha diversity by mean ASV richness and Beta diversity by unweighed UNIFRAC (PERMANOVA p. 0.004 q. 0.004) PCoA of fecal 16s rRNA sequencing of stool from 13-week-old Cre ERT-carrying or Cre-ERT deficient, TMX treated male animals. Volcano plots of differential abundance testing of fecal microbiota species (sequence variants) from samples in (B). FDR-corrected statistically significant species are color highlighted.

Supplementary Figure 10: Effects of antibiotic treatment on weight and metabolic parameters. Weight curves of HFD-fed mice on vehicle (Veh) or antibiotic treatment (ampicillin, neomycin, vancomycin and metronidazole). WT animals **(A)** and EEC$^{\Delta Col}$ mice **(B)** are shown. Mean values are represented (dot) and error bars are ± S.E.M. Two-tailed unpaired t-test used for all pairwise comparisons. **p<0.01, ***p<0.001.

Supplementary Figure 11: Fecal L-glutamic acid in various obesity models. **(A)** and **(B)** Weight curves and food intake in 4 groups of mice subjected to TMX treatment or not for inducible EEC deletion (relates to Figure 4D). **(C)** Fecal L-glutamic acid concentration in 8-week-old 4h-fasted WT mice in specific pathogen free (SPF) or GF conditions. **(D)** Fecal L-glutamic acid concentration from 13-week-old WT or iEEC$^{\Delta Col}$ mice in fasting (6 hours) and refed conditions (1 hour after refeeding). **(E)** and **(F)** Weight of 10-week-old (E) and 16-week-old (F) WT or ob/ob male mice. **(G)** Fecal L-glutamic acid concentration of 10-week-old 4h fasted WT or ob/ob male animals. Mean values (dot) and error bars are presented in (A) and (B) , while mean values (bars) and individual values are shown by jitter plots in (C-G). Error bars are ± S.E.M. ANOVA (panels A and B) or two-tailed unpaired t-test were used for group comparisons (C-G). *p<0.05, **p<0.01, ***p<0.001, ****p<0.0001.

Figure S1

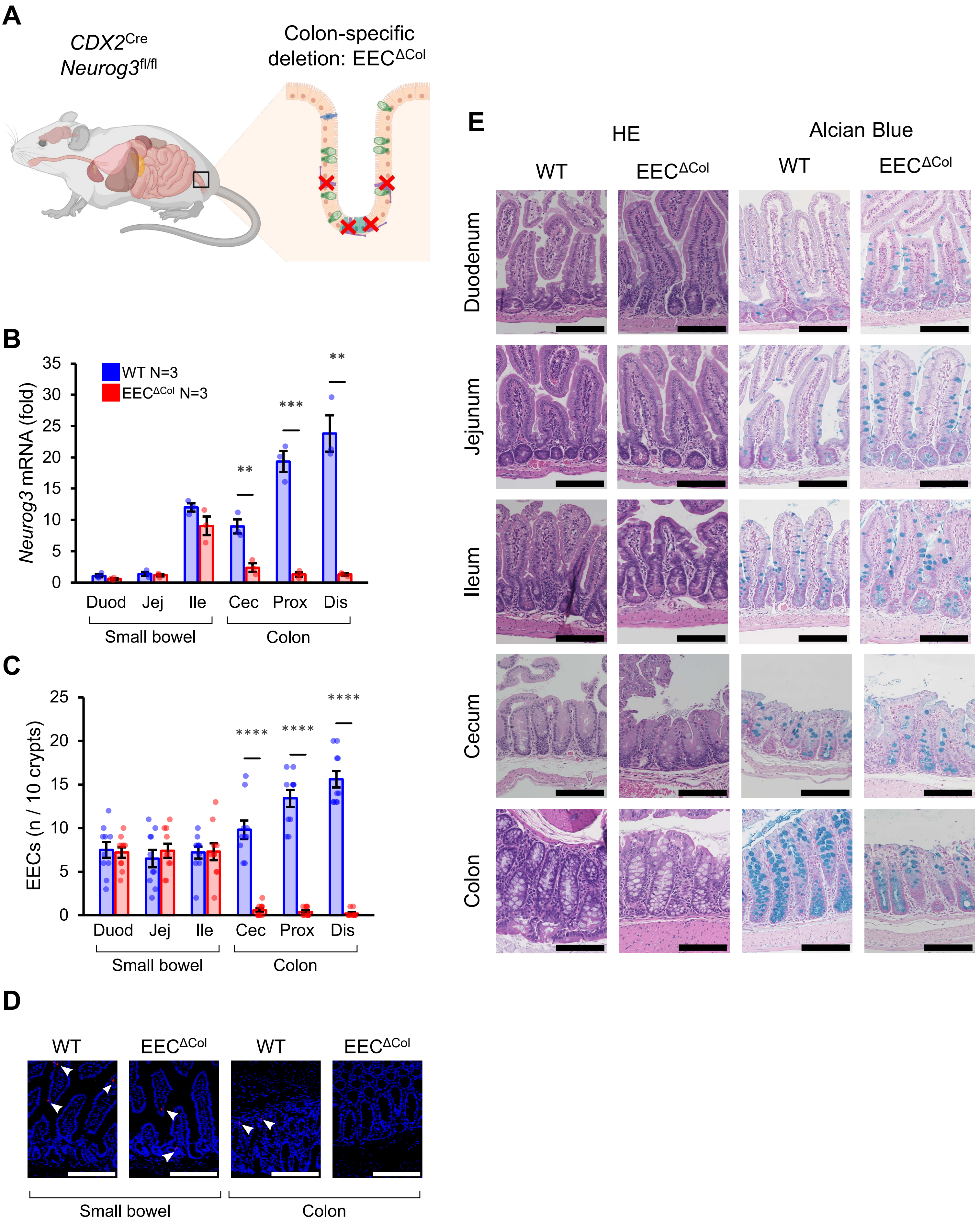

A
CDX2Cre
Neurog3fl/fl
Colon-specific deletion: EECΔCol
B
WT N=3
EECΔCol N=3
Neurog3 mRNA (fold)
Duod
Jej
Ile
Cec
Prox
Dis
Small bowel
Colon
C
EECs (n / 10 crypts)
D
WT
EECΔCol
E
HE
Alcian Blue
Duodenum
Jejunum
Ileum
Cecum
Colon

Figure S2

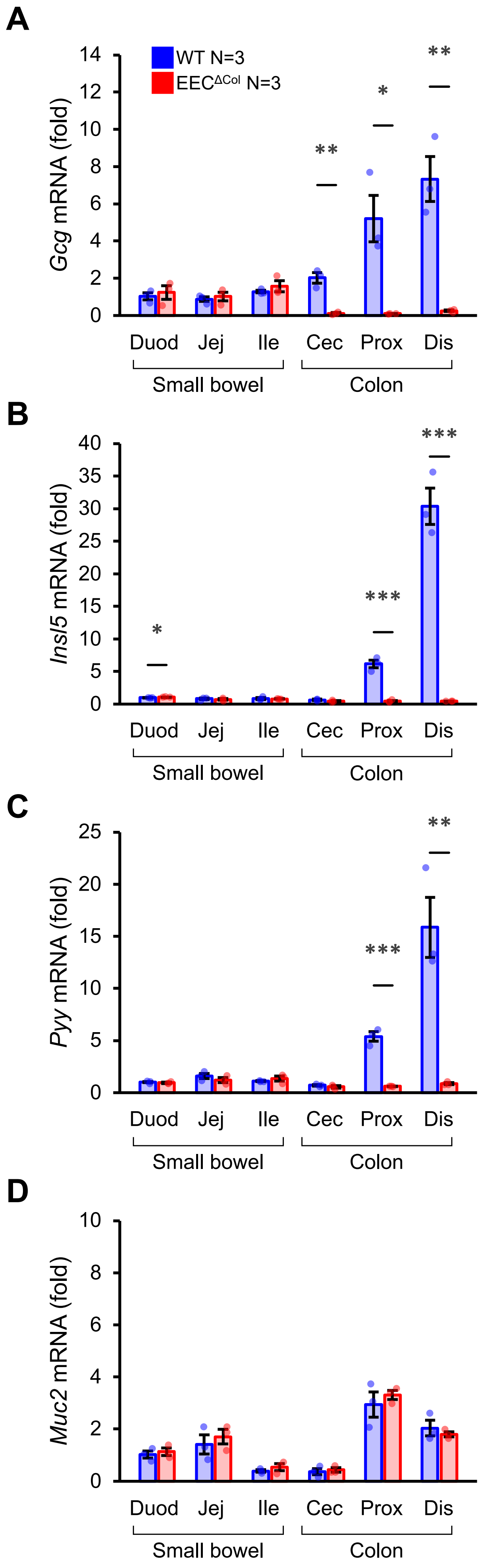
A
WT N=3
EEC^ΔCol N=3
Gcg mRNA (fold)
14
12
10
8
6
4
2
0
**
*
**
Duod
Jej
Ile
Cec
Prox
Dis
Small bowel
Colon
B
Insl5 mRNA (fold)
40
35
30
25
20
15
10
5
0
*
***
***
Duod
Jej
Ile
Cec
Prox
Dis
Small bowel
Colon
C
Pyy mRNA (fold)
25
20
15
10
5
0
***
**
Duod
Jej
Ile
Cec
Prox
Dis
Small bowel
Colon
D
Muc2 mRNA (fold)
10
8
6
4
2
0
Duod
Jej
Ile
Cec
Prox
Dis
Small bowel
Colon

Figure S3

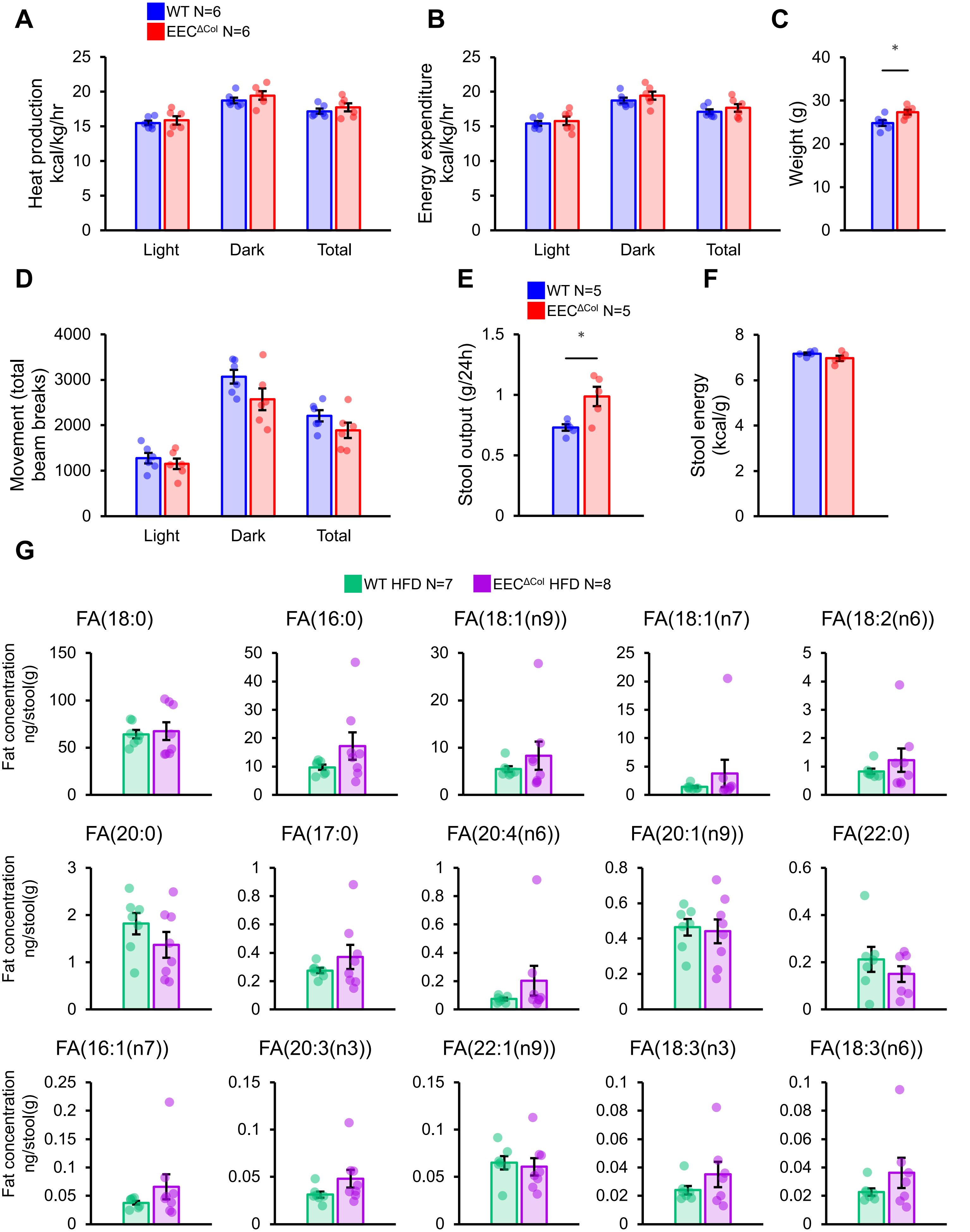

A
WT N=6
EEC$^{\Delta Col}$ N=6
Heat production kcal/kg/hr
25
20
15
10
5
0
Light
Dark
Total
B
Energy expenditure kcal/kg/hr
C
Weight (g)
40
30
20
10
*
D
Movement (total beam breaks)
4000
3000
2000
1000
E
WT N=5
EEC$^{\Delta Col}$ N=5
Stool output (g/24h)
1.5
1
0.5
*
F
Stool energy (kcal/g)
8
6
4
2
G
WT HFD N=7
EEC$^{\Delta Col}$ HFD N=8
Fat concentration ng/stool(g)
FA(18:0)
FA(16:0)
FA(18:1(n9))
FA(18:1(n7)
FA(18:2(n6))
FA(20:0)
FA(17:0)
FA(20:4(n6))
FA(20:1(n9))
FA(22:0)
FA(16:1(n7))
FA(20:3(n3))
FA(22:1(n9))
FA(18:3(n3)
FA(18:3(n6))

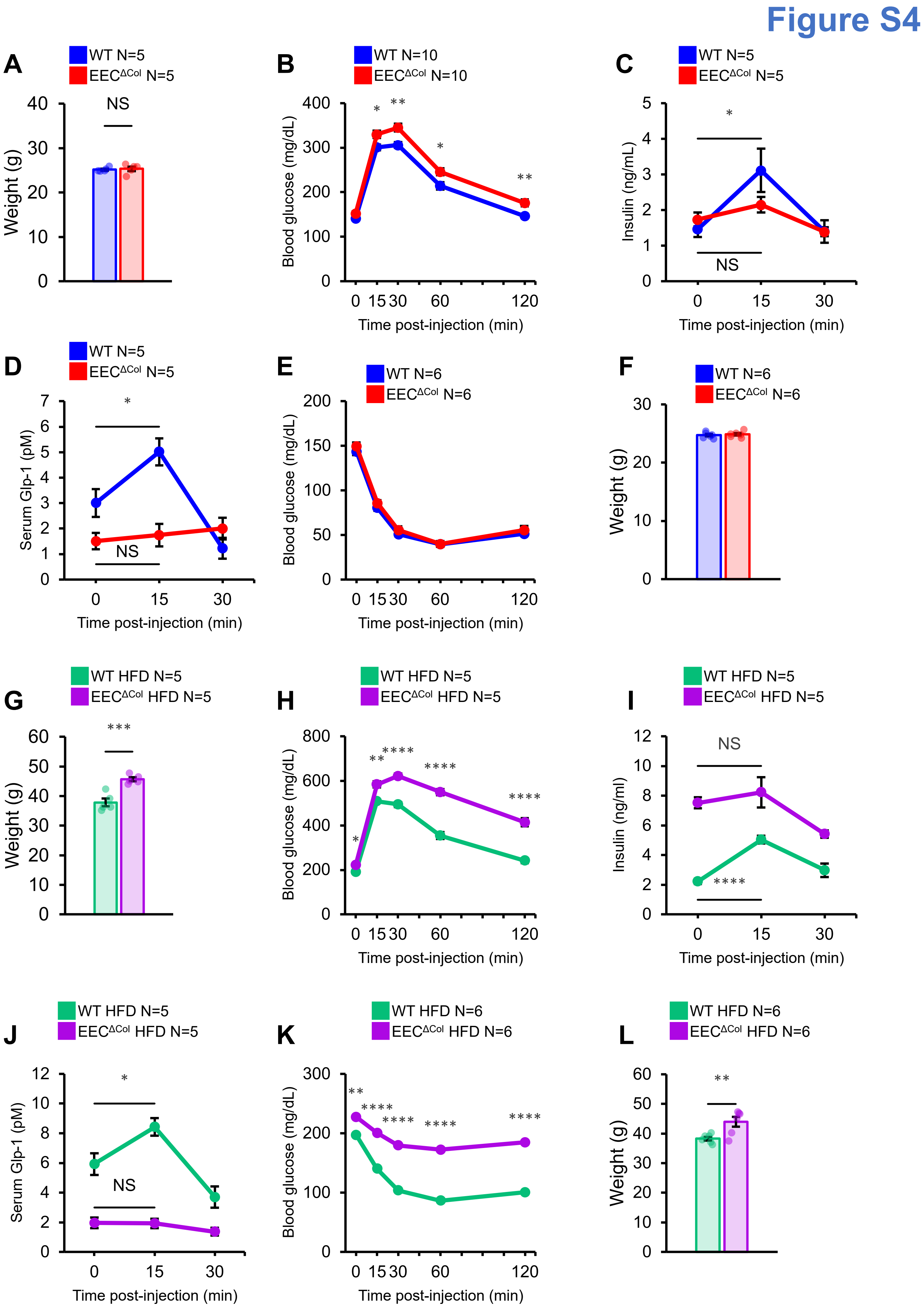

Figure S4
A
WT N=5
EEC$^{\Delta Col}$ N=5
NS
Weight (g)
B
WT N=10
EEC$^{\Delta Col}$ N=10
Blood glucose (mg/dL)
Time post-injection (min)
C
WT N=5
EEC$^{\Delta Col}$ N=5
Insulin (ng/mL)
NS
Time post-injection (min)
D
WT N=5
EEC$^{\Delta Col}$ N=5
Serum Glp-1 (pM)
NS
Time post-injection (min)
E
WT N=6
EEC$^{\Delta Col}$ N=6
Blood glucose (mg/dL)
Time post-injection (min)
F
WT N=6
EEC$^{\Delta Col}$ N=6
Weight (g)
G
WT HFD N=5
EEC$^{\Delta Col}$ HFD N=5
Weight (g)
H
WT HFD N=5
EEC$^{\Delta Col}$ HFD N=5
Blood glucose (mg/dL)
Time post-injection (min)
I
WT HFD N=5
EEC$^{\Delta Col}$ HFD N=5
NS
Insulin (ng/ml)
Time post-injection (min)
J
WT HFD N=5
EEC$^{\Delta Col}$ HFD N=5
Serum Glp-1 (pM)
NS
Time post-injection (min)
K
WT HFD N=6
EEC$^{\Delta Col}$ HFD N=6
Blood glucose (mg/dL)
Time post-injection (min)
L
WT HFD N=6
EEC$^{\Delta Col}$ HFD N=6
Weight (g)

Figure S5

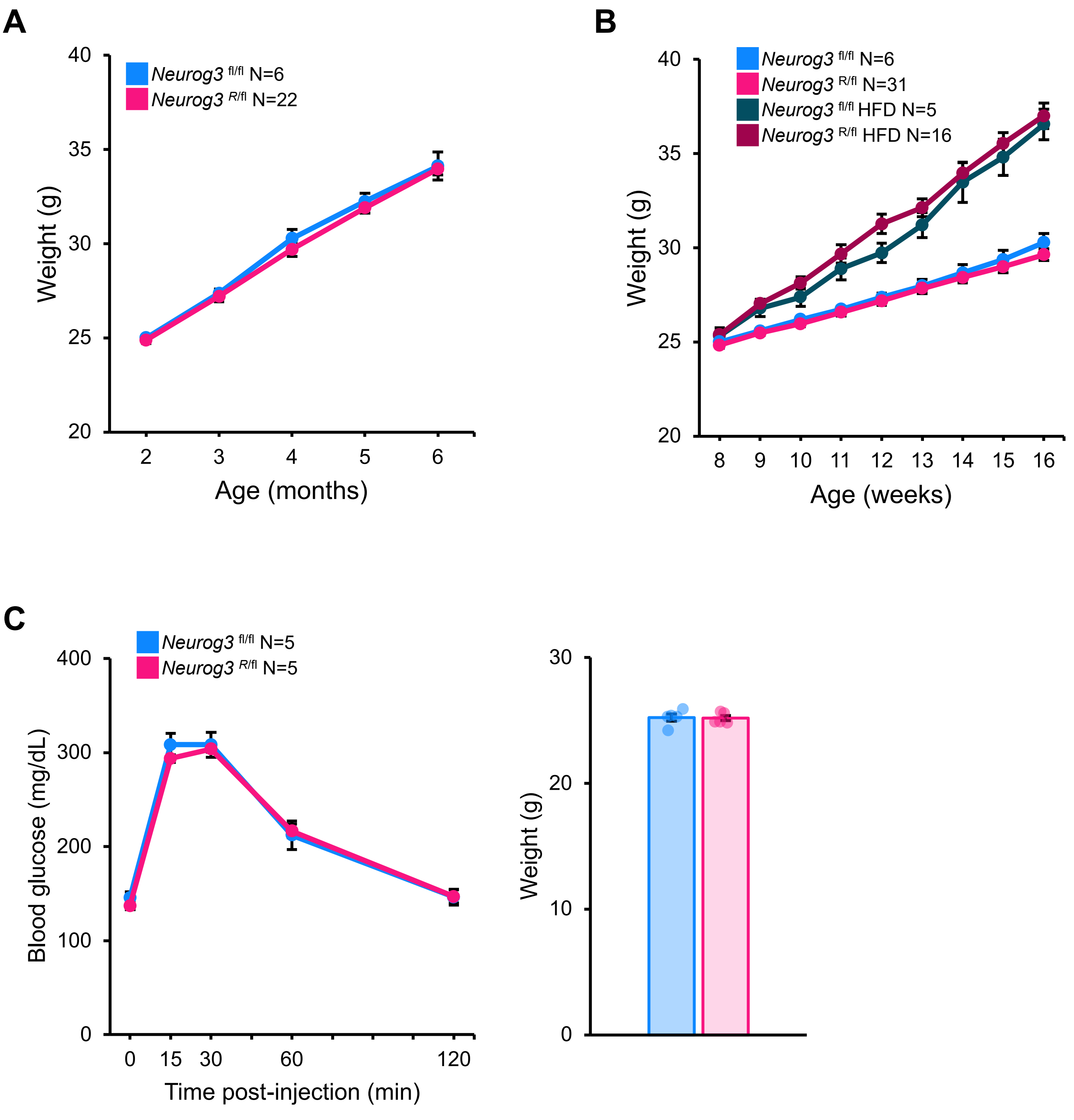
A
Neurog3 fl/fl N=6
Neurog3 R/fl N=22
Weight (g)
40
35
30
25
20
2
3
4
5
6
Age (months)
B
Neurog3 fl/fl N=6
Neurog3 R/fl N=31
Neurog3 fl/fl HFD N=5
Neurog3 R/fl HFD N=16
Weight (g)
40
35
30
25
20
8
9
10
11
12
13
14
15
16
Age (weeks)
C
Neurog3 fl/fl N=5
Neurog3 R/fl N=5
Blood glucose (mg/dL)
400
300
200
100
0
0
15
30
60
120
Time post-injection (min)
Weight (g)
30
20
10
0

Figure S6

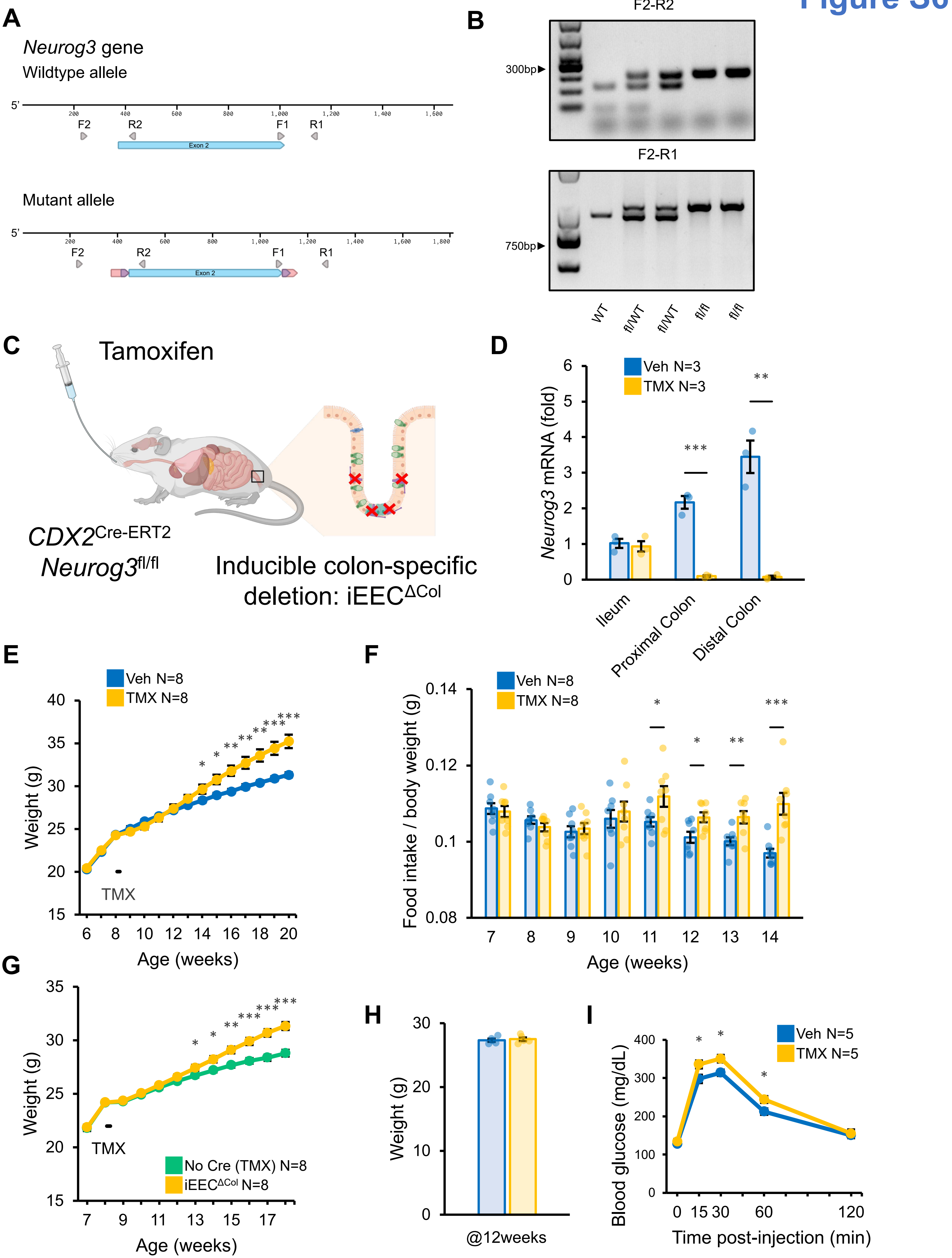
A
Neurog3 gene
Wildtype allele
F2
R2
F1
R1
Exon 2
Mutant allele
B
F2-R2
300bp
F2-R1
750bp
WT
fl/WT
fl/WT
fl/fl
fl/fl
C
Tamoxifen
CDX2Cre-ERT2
Neurog3fl/fl
Inducible colon-specific deletion: iEECΔCol
D
Veh N=3
TMX N=3
Neurog3 mRNA (fold)
Ileum
Proximal Colon
Distal Colon
E
Veh N=8
TMX N=8
Weight (g)
TMX
Age (weeks)
F
Veh N=8
TMX N=8
Food intake / body weight (g)
Age (weeks)
G
Weight (g)
TMX
No Cre (TMX) N=8
iEECΔCol N=8
Age (weeks)
H
Weight (g)
@12weeks
I
Veh N=5
TMX N=5
Blood glucose (mg/dL)
Time post-injection (min)

Figure S7

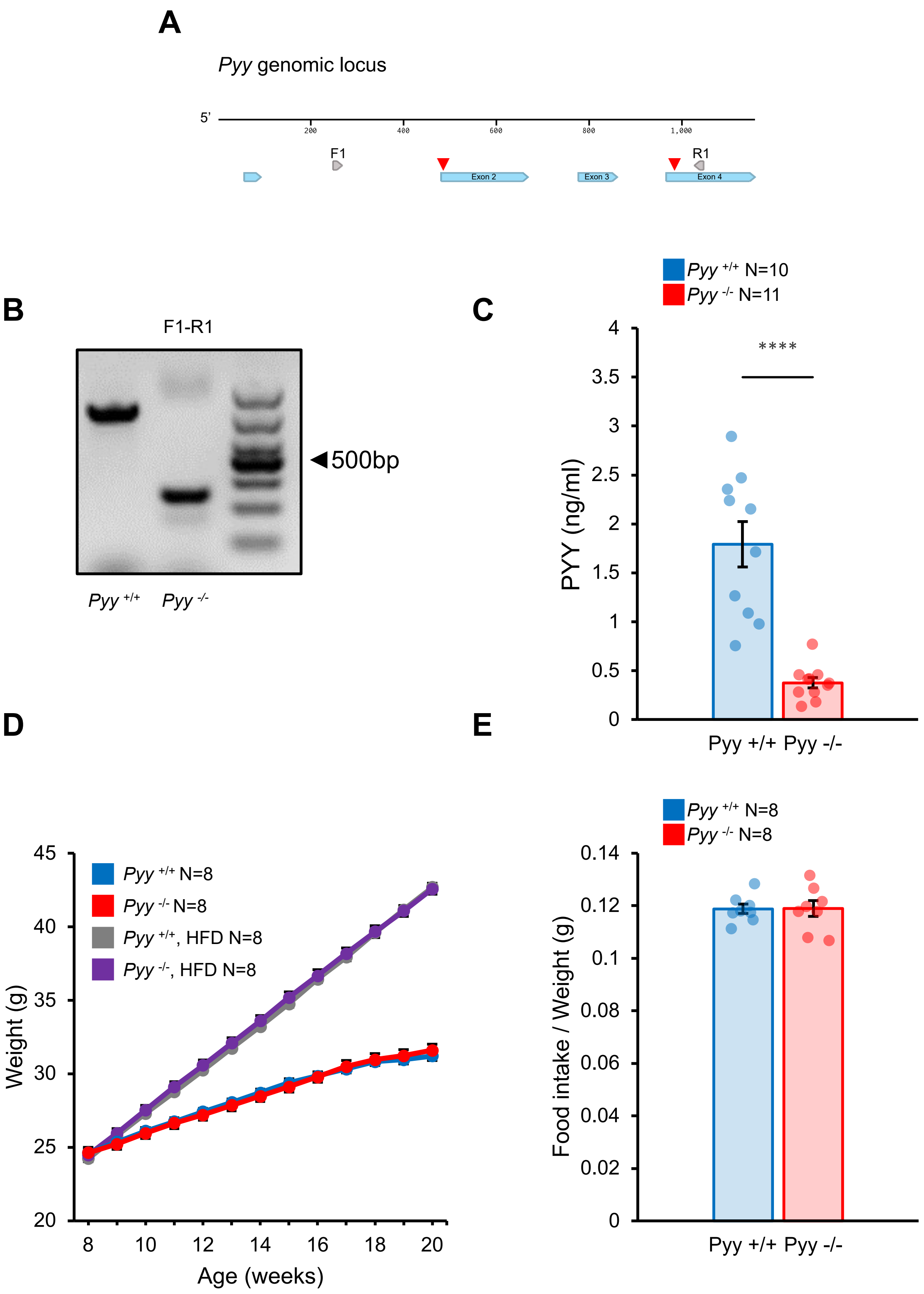
A
Pyy genomic locus
5'
200
400
600
800
1,000
F1
R1
Exon 2
Exon 3
Exon 4
B
F1-R1
◀500bp
Pyy +/+
Pyy -/-
C
Pyy +/+ N=10
Pyy -/- N=11
PYY (ng/ml)
****
Pyy +/+ Pyy -/-
D
Pyy +/+ N=8
Pyy -/- N=8
Pyy +/+, HFD N=8
Pyy -/-, HFD N=8
Weight (g)
Age (weeks)
E
Pyy +/+ N=8
Pyy -/- N=8
Food intake / Weight (g)
Pyy +/+ Pyy -/-

Figure S8

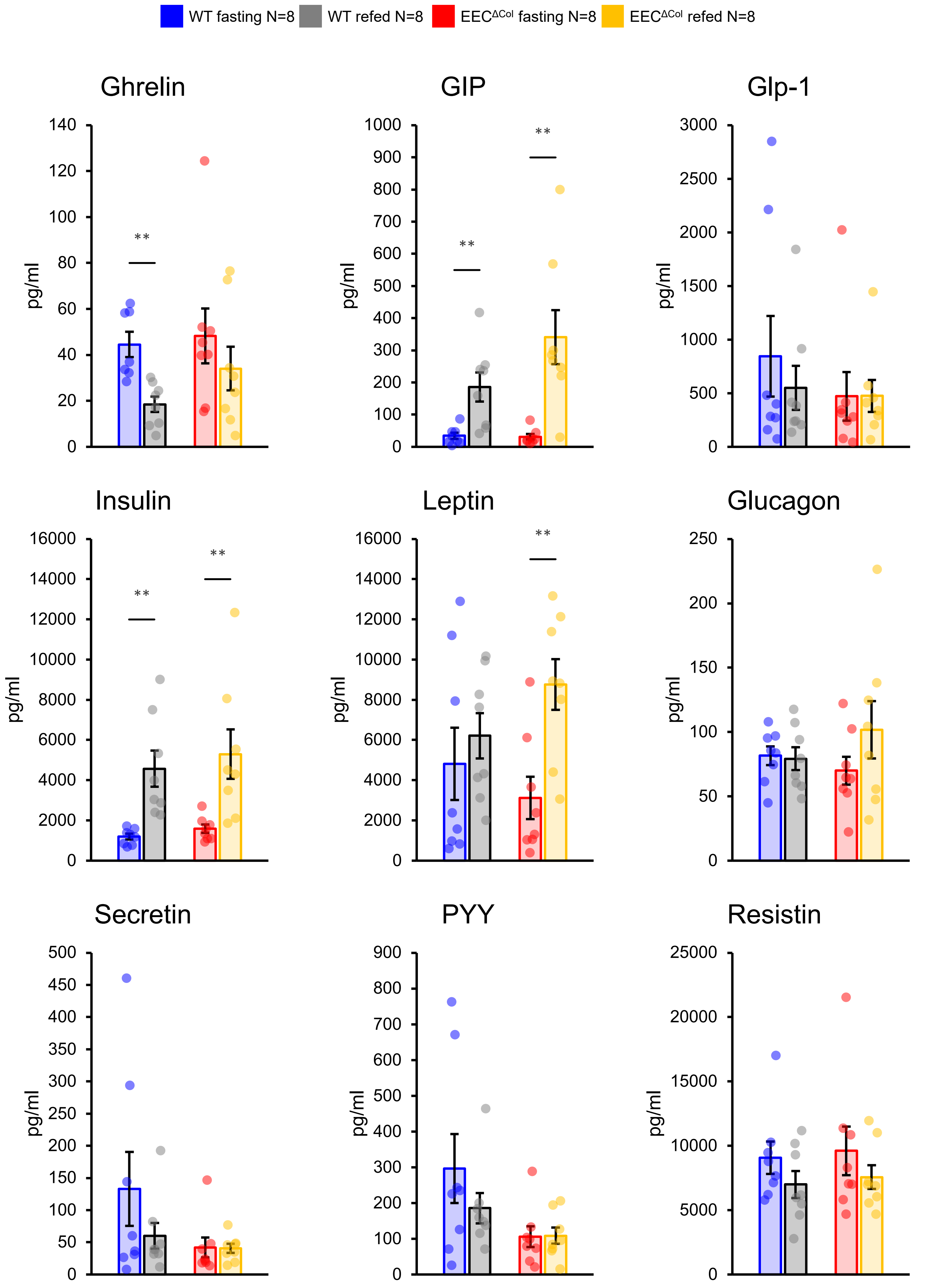

WT fasting N=8
WT refed N=8
EEC$^{\Delta Col}$ fasting N=8
EEC$^{\Delta Col}$ refed N=8
Ghrelin
GIP
Glp-1
Insulin
Leptin
Glucagon
Secretin
PYY
Resistin
pg/ml
**

Figure S9

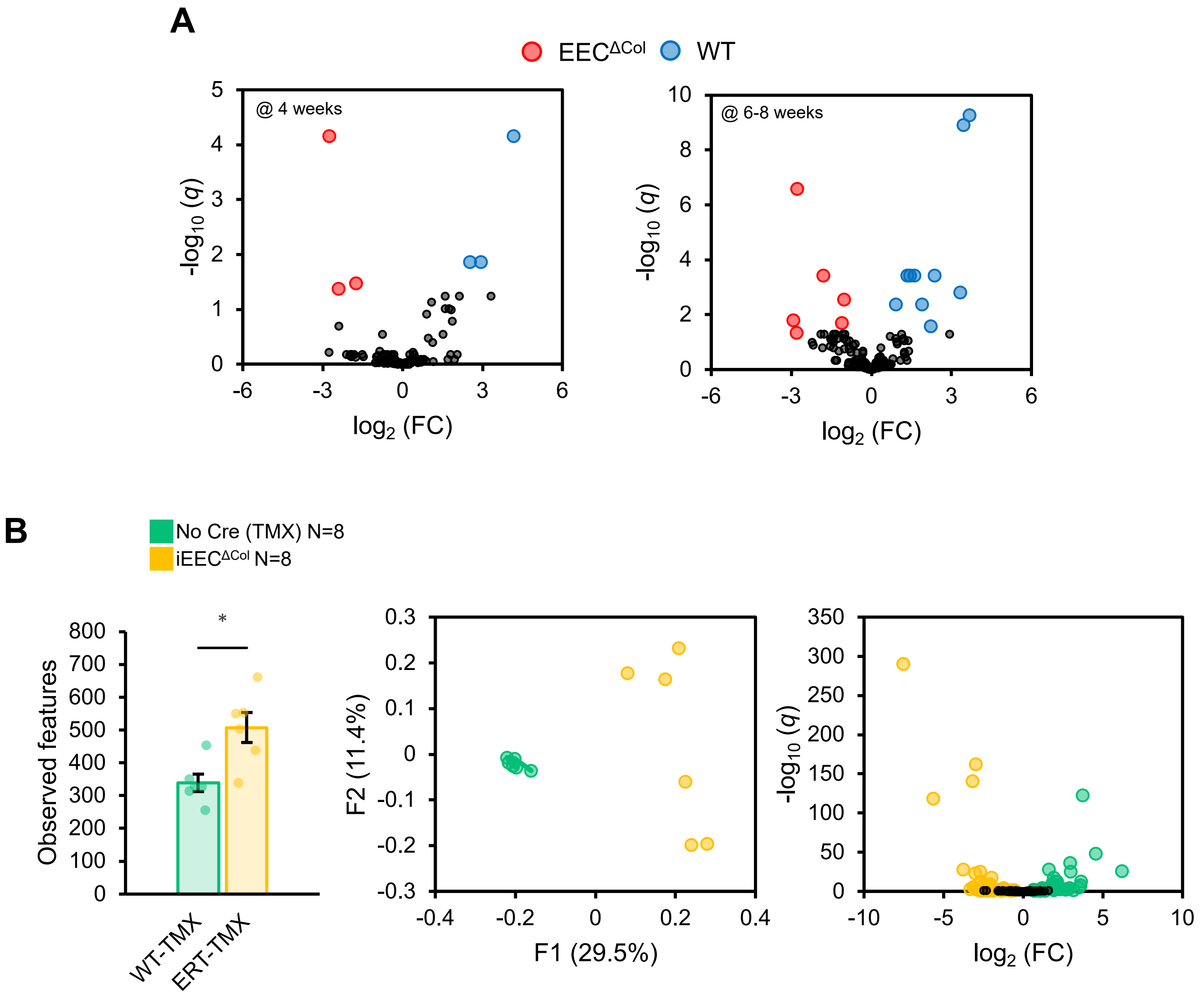
A
EECΔCol
WT
@ 4 weeks
@ 6-8 weeks
-log10 (q)
log2 (FC)
B
No Cre (TMX) N=8
iEECΔCol N=8
Observed features
*
WT-TMX
ERT-TMX
F1 (29.5%)
F2 (11.4%)
-log10 (q)
log2 (FC)

Figure S10

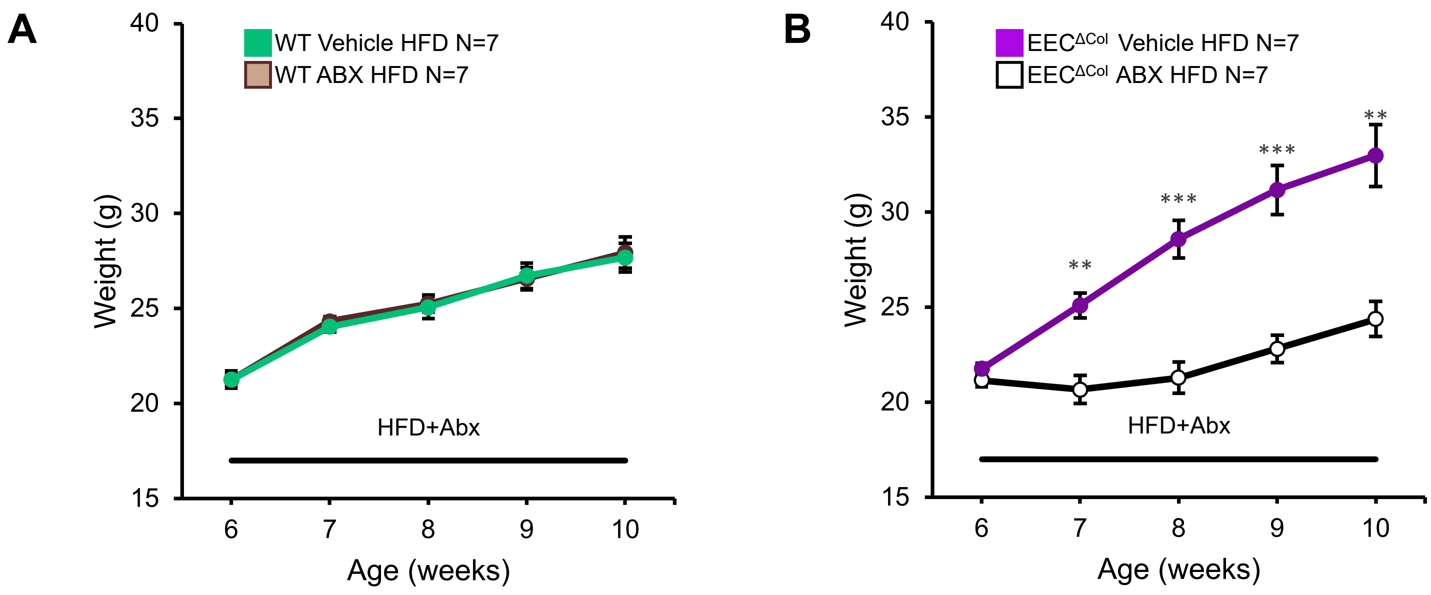
A
WT Vehicle HFD N=7
WT ABX HFD N=7
Weight (g)
40
35
30
25
20
15
HFD+Abx
6
7
8
9
10
Age (weeks)
B
EEC$^{\Delta Col}$ Vehicle HFD N=7
EEC$^{\Delta Col}$ ABX HFD N=7
Weight (g)
40
35
30
25
20
15
**
***
***
**
HFD+Abx
6
7
8
9
10
Age (weeks)

Figure S11

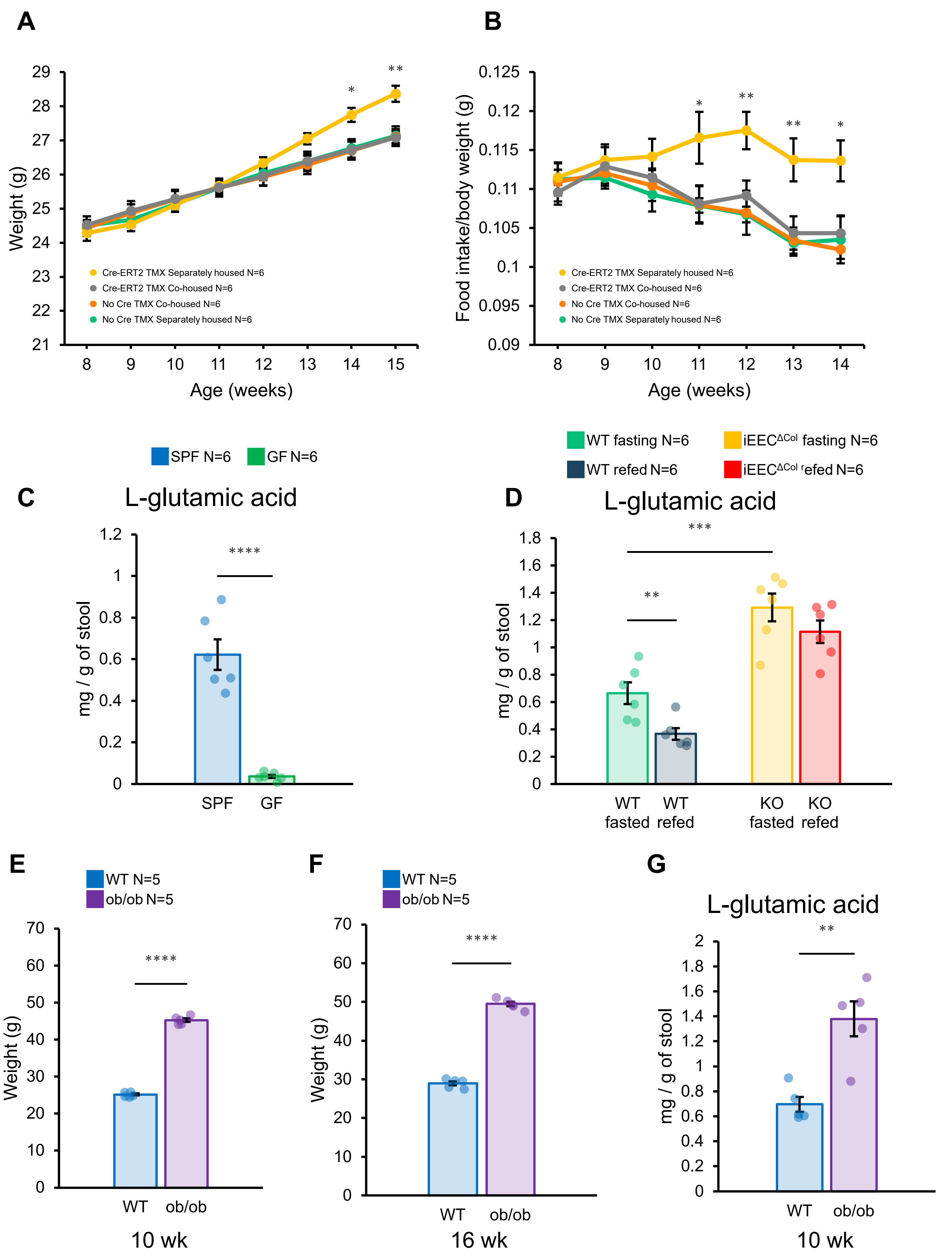
A
Weight (g)
Age (weeks)
Cre-ERT2 TMX Separately housed N=6
Cre-ERT2 TMX Co-housed N=6
No Cre TMX Co-housed N=6
No Cre TMX Separately housed N=6
B
Food intake/body weight (g)
Age (weeks)
Cre-ERT2 TMX Separately housed N=6
Cre-ERT2 TMX Co-housed N=6
No Cre TMX Co-housed N=6
No Cre TMX Separately housed N=6
SPF N=6
GF N=6
C
L-glutamic acid
mg / g of stool
SPF
GF
WT fasting N=6
iEEC^ΔCol fasting N=6
WT refed N=6
iEEC^ΔCol refed N=6
D
L-glutamic acid
mg / g of stool
WT fasted
WT refed
KO fasted
KO refed
E
WT N=5
ob/ob N=5
Weight (g)
WT
ob/ob
10 wk
F
WT N=5
ob/ob N=5
Weight (g)
WT
ob/ob
16 wk
G
L-glutamic acid
mg / g of stool
WT
ob/ob
10 wk
